# Noncanonical roles of membranous lysyl-tRNA synthetase in transducing cell-substrate signaling for invasive dissemination of colon cancer spheroids in 3D collagen I gels

**DOI:** 10.18632/oncotarget.4130

**Published:** 2015-05-12

**Authors:** Seo Hee Nam, Doyeun Kim, Mi-Sook Lee, Doohyung Lee, Tae Kyoung Kwak, Minkyung Kang, Jihye Ryu, Hye-Jin Kim, Haeng Eun Song, Jungeun Choi, Gyu-Ho Lee, Sang-Yeob Kim, Song Hwa Park, Dae Gyu Kim, Nam Hoon Kwon, Tai Young Kim, Jean Paul Thiery, Sunghoon Kim, Jung Weon Lee

**Affiliations:** ^1^ Interdisciplinary Program in Genetic Engineering, Seoul National University, Seoul, Republic of Korea; ^2^ Department of Pharmacy, Medicinal Bioconvergence Research Center, Research Institute of Pharmaceutical Sciences, College of Pharmacy, Seoul National University, Seoul, Republic of Korea; ^3^ Department of Biomedical Sciences, College of Medicine, Seoul National University, Seoul, Republic of Korea; ^4^ Department of Medicine, University of Ulsan, College of Medicine, Seoul, Republic of Korea; ^5^ Cancer Science Institute, National University of Singapore, Singapore; ^6^ Institute of Molecular and Cell Biology, A*STAR, Singapore; ^7^ Department of Biochemistry, School of Medicine, National University of Singapore, Singapore

**Keywords:** 3D culture, cancer metastasis, cell-ECM adhesion, lysyl-tRNA synthetase, signal transduction

## Abstract

The adhesion properties of cells are involved in tumor metastasis. Although KRS at the plasma membrane is shown important for cancer metastasis, additionally to canonical roles of cytosolic KRS in protein translation, how KRS and its downstream effectors promote the metastatic migration remains unexplored. Disseminative behaviors (an earlier metastatic process) of colon cancer cell spheroids embedded in 3D collagen gels were studied with regards to cell adhesion properties, and relevance in KRS^−/+^ knocked-down animal and clinical colon cancer tissues. Time-lapse imaging revealed KRS-dependent cell dissemination from the spheroids, whereas KRS-suppressed spheroids remained static due to the absence of outbound movements supported by cell-extracellular matrix (ECM) adhesion. While keeping E-cadherin at the outward disseminative cells, KRS caused integrin-involved intracellular signaling for ERK/c-Jun, paxillin, and cell-ECM adhesion-mediated signaling to modulate traction force for crawling movement. KRS-suppressed spheroids became disseminative following ERK or paxillin re-expression. The KRS-dependent intracellular signaling activities correlated with the invasiveness in clinical colon tumor tissues and in KRS^−/+^ knocked-down mice tissues. Collectively, these observations indicate that KRS at the plasma membrane plays new roles in metastatic migration as a signaling inducer, and causes intracellular signaling for cancer dissemination, involving cell-cell and cell-ECM adhesion, during KRS-mediated metastasis.

## INTRODUCTION

Cancer metastasis is a complex process involving multiple steps, such as dissemination from the primary tumor mass, migration, and invasion, which occur even before the intravasation processes [1]. Dissemination from the primary tumor mass facilitates metastatic cancer cell migration toward distal sites, and, along the way, their invasion through the stroma to reach vessels or lymph nodes [2]. In addition to strategies aimed at the regression of the primary tumor mass through chemoprevention and/or radiation, anti-metastatic strategies that block this dissemination process, at an early stage of metastasis, would be clinically important for achieving a higher cure rate for cancer.

Numerous signaling or adaptor molecules have been shown to play critical roles in facilitating cancer cells to metastasize. Of these molecules, we are interested in revealing the roles of aminoacyl-tRNA synthetases in tumorigenesis and metastasis [3]. Cytosolic aminoacyl-tRNA synthetases are traditionally and importantly involved in protein translation. However, lysyl-tRNA synthetase (KRS) was recently shown to function in immune responses [4, 5] as well as in tumor metastasis [6, 7], suggesting additional biological functions beyond protein translation for this protein. KRS is phosphorylated at the Thr52 residue by p38MAPK, which causes it to dissociate from the cytosolic multi-tRNA synthetase complex and translocate to the plasma membrane, where it associates with and stabilizes a 67 kDa laminin receptor (p67LR) involved in migration and metastasis [6]. p67LR is known to associate with integrin α6β1 [8] or α6β4 [9], which have roles in cell adhesion to the ECM and in tumor cell invasion [10]. The association of KRS with p67LR is sufficiently targeted to allow the inhibition of KRS-dependent metastasis in a subcutaneously cell-injected animal model, suggesting that KRS is a promising target for anti-cancer metastatic strategies [7]. These previous studies have focuses on the regulatory processes only upstream of KRS, that is, to see how KRS is regulated for migratory functions. However, the molecules downstream of KRS at plasma membrane have never been explored.

Therefore, as part of our efforts to develop anti-metastatic therapeutics by targeting an earlier metastatic process, here we report that KRS may cause cell dissemination from spheroids embedded in an ECM-surrounded 3-dimensional (3D) environment. In addition, KRS appears to play regulatory roles in cell adhesion by transducing intracellular signaling to activate ERK1/2 and paxillin during cancer cell dissemination from primary tumor masses. This 3D gel system would be beneficial if the observed dissemination process could be mechanistically related to KRS-dependent dissemination in *in vivo*-like circumstances and could also generally be useful as a template screening system for anti-metastasis reagents.

## RESULTS

### KRS-dependent dissemination of HCT116 spheroids in 3D collagen I gels

**Figure 1:**
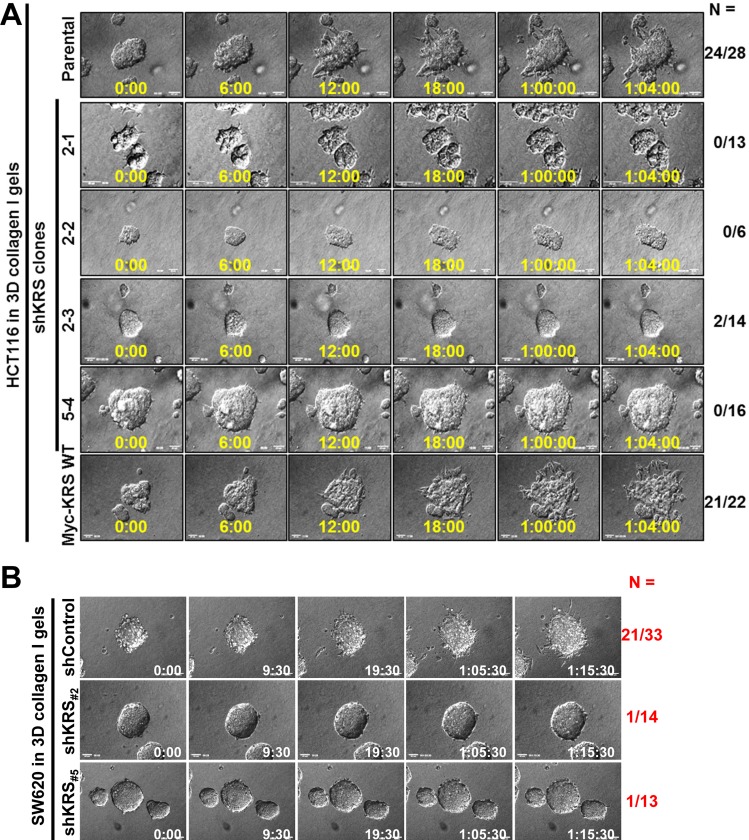

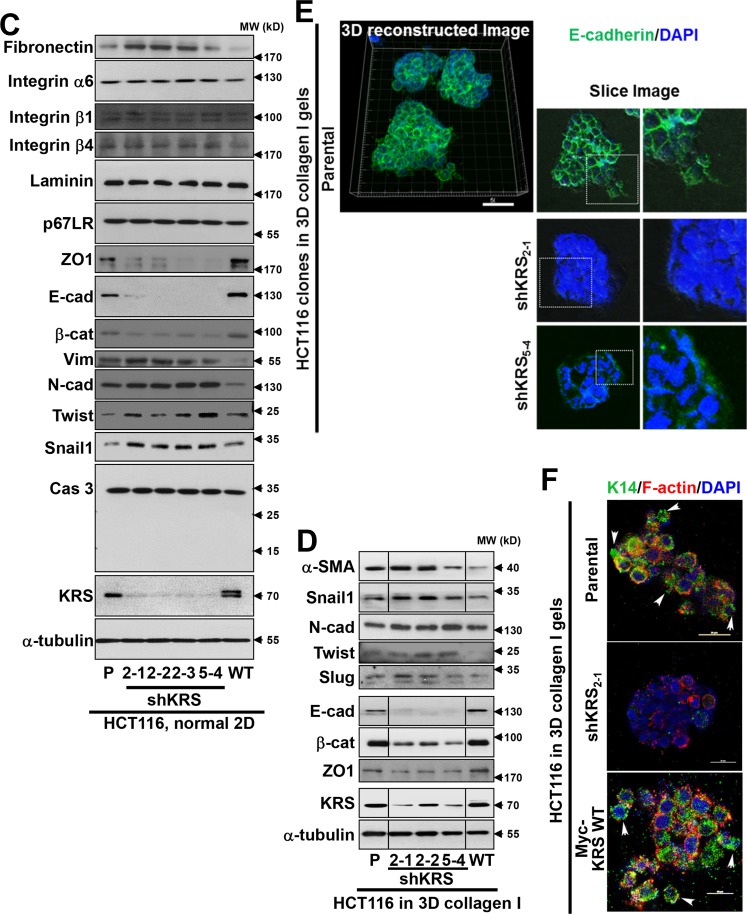
KRS-dependent dissemination of HCT116 spheroids in 3D collagen I gels (**A** and **B**) Spheroids of HCT116 (A, parental, stable shKRS-transfectant clones of 2-1, 2-2, 2-3, and 5-4, and stably myc-KRS WT overexpressing cells) or SW620 (B, shControl, #2 and #5 for stable shKRS clones) colon cancer cells embedded in 3D collagen I gels were imaged by time-lapse microscopy for 28 h (1 day 4 h; 1:04:00) or 40 h (1:16:00). The numbers to the right of the figure in the ‘a/b’ format depict disseminative phenotype cases/total experimental cases. See also Movie S1 to S9. (**C** and **D**) Standard western blots for the indicated epithelial or mesenchymal markers from KRS-expressing parental HCT116 (P), stably myc-KRS wildtype-overexpressing HCT116 (KRS WT), and stably KRS-suppressed HCT116 cell clones (2-1, 2-2, 2-3, and 5-4 for shKRS) normally cultured in 2D 10% FBS-containing condition (**C**) or embedded in 3D collagen I gels for 24 h (**D**). (**E**) Spheroids of HCT116 parental cells (*top*) or HCT116 shKRS cells (2-1 clone, *middle*), and HCT116-KRS WT (*bottom*) cells embedded in 3D collagen I gels for 24 h were double-stained with DAPI (blue) and anti-E-cadherin (green) antibody. Note that disseminated small group of KRS-expressing cells were still E-cadherin-positive, whereas KRS-suppressed cells were negative for E-cadherin and did not disseminate. Three-dimensionally reconstructed image or slice views from confocal live images are shown. (**F**) Cells were embedded in 3D collagen I gels for 24 h and then fixed, permeabilized, and immunostained for K14 (green) in parallel with phalloidin (red) and DAPI (blue). Arrow heads depict cells presumably disseminating with K14 expression. Data represent three independent experiments. See also Figure S1.

We questioned whether KRS could be important for cell dissemination from tumor masses, and sought to monitor HCT116 spheroids (with various KRS expression levels) embedded into 3D collagen I gels using time-lapse photography. Cellular spheroids, with diameters between 70 and 100 μm, were sieved and embedded into collagen I (2.0 mg/ml) gels. We found that KRS-expressing cells exhibited outbound movement or dissemination of single or small groups of cells (Figure 1A, Movies S1 and S6), whereas KRS-suppressed cell clones did not (Figure 1A, Movies S2 to S5). Another colon cancer SW620 cell line also exhibited KRS-dependent dissemination from spheroids (Figure 1B, Movies S7 to S9).

Furthermore, epithelial protein markers including E-cadherin were expressed at higher levels in KRS-expressing parental and KRS-overexpressing (i.e., KRS-positive) HCT116 cells, whereas KRS-suppressed cell clones (Figure S1A) decreased E-cadherin, β-catenin, and ZO-1 expression and concomitantly enhanced the expression of mesenchymal markers, such as snail1, twist, vimentin, N-cadherin, and fibronectin (Figure 1C). When we checked whether KRS knockdown could affect general protein translations, we found no difference between KRS-expressing and KRS-suppressed cell clone (Figure S1B). Furthermore, KRS-suppressed cell clones showed no change in the expression of laminin, p67-laminin receptor (p67LR), integrin chains α6, β1, or β4 as compared with KRS-expressing cells (Figure 1C).

KRS-overexpression causes stabilization of p67LR on the membrane upon laminin treatment [6]. Thus we checked whether the laminin-induced increase in 67LR levels was different between KRS-positive and KRS-suppressed HCT116 cells. Unlike KRS-positive cells, which were sensitive to laminin treatment for p67LR stabilization, KRS-suppressed cells were insensitive to the laminin treatment (Figure S1C).

Furthermore, KRS-suppressed HCT116 cells showed increased mesenchymal markers and decreased epithelial markers 24 h after embedding (Figure 1D); however, these cells did not disseminate (Figure 1A). This lack of dissemination might be related to the partial EMT phenotypes of the cells, and suggests that the loss of cell-cell junction molecules may not be all that is required for dissemination. Consistently, KRS-expressing parental cells showed disseminative margins that were positive for E-cadherin expression, whereas KRS-suppressed cells showed neither (Figure 1E). When spheroids were stained for another epithelial marker (cytokeratin 14, K14), KRS-expressing parental or -overexpressing cells at the disseminative margins were positive for K14, whereas KRS-suppressed cells were negative (Figure 1F).

### KRS-dependent cell-substrate adhesion

**Figure 2:**
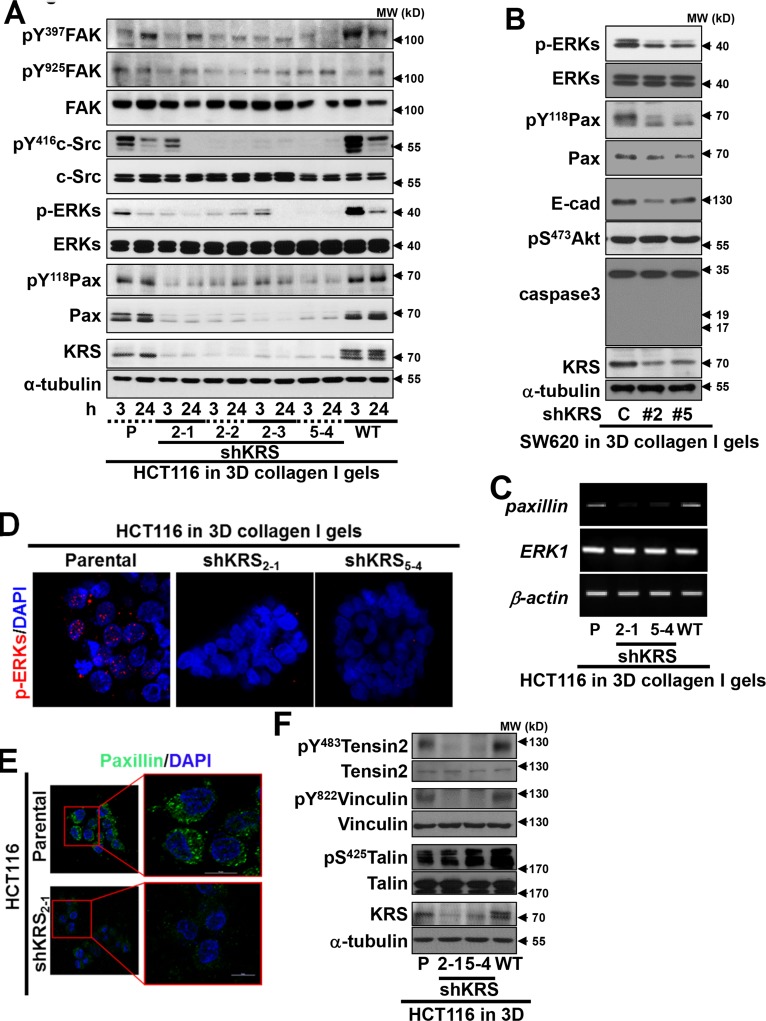
KRS-dependent cell-laminin adhesion (**A** to **C**) HCT116 (A and C) or SW620 (B, #2 and #5 for stable shKRS clones) cells embedded in 3D collagen I gels for 3 or 24 h (A, B, and C) were harvested for whole-cell extracts prior to performing standard western blots for the indicated molecules (**A** and **B**) or RT-PCR (**C**). (**D** and **E**) Cell spheroids embedded in 3D collagen I gels for 24 h were immunostained for pERK1/2 (D, red) or paxillin (E, green), with DAPI (blue) co-staining. Right panels are enlarged from the red boxes in the corresponding left panels (**E**). (**F**) Cells embedded in 3D collagen I gels for 24 h were harvested and processed for standard western blots for the molecules. Data represent three independent experiments.

**Figure 3:**
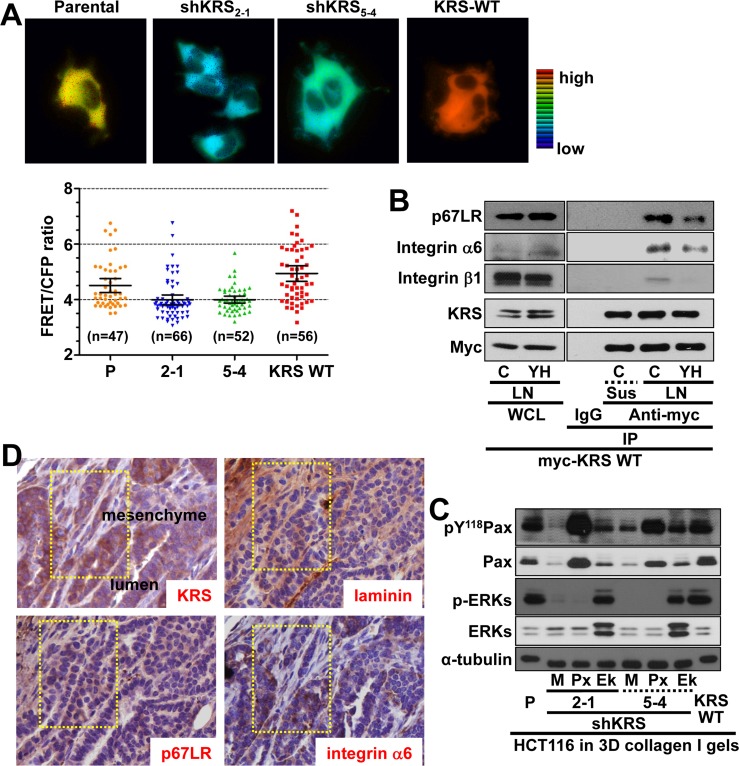

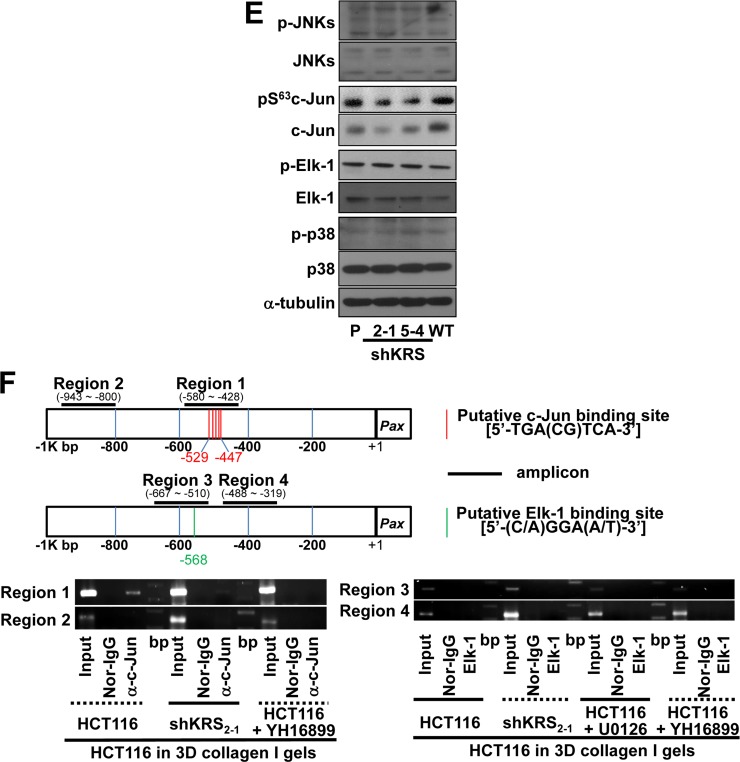
KRS-mediated formation of a p67LR/integrin α6β1 complex for ERK1/2 activation (**A**) Cells reseeded onto laminin-precoated cover glasses for 24 h were analyzed for ERK activity using an ERK FRET biosensor. The mean fluorescence intensities of each cell from separate cell images (n= separately imaged cell numbers) were analyzed using the MetaMorph software and are presented as the mean ± standard deviation (SD) of the FRET value/CFP ratio. See also Figure S2 and Movie S9 to S10. (**B**) Myc-KRS WT cells kept in suspension (Sus), or reseeded onto laminin (LN)-precoated dishes within 2% FBS-containing media in the presence of DMSO (**C**) or YH16899 (YH) treatment were harvested for whole cell lysates (WCL) prior to immunoprecipitation using normal IgG (IgG) or anti-myc antibody along with standard western blotting for the indicated molecules. (**C**) Spheroids prepared using KRS-expressing parental (P), KRS WT-overexpressing cells, and KRS-suppressed cells transiently transfected with mock (M), paxillin (Px), or ERK1/2 (Ek) expression vectors for 24 h were embedded into 3D collagen I gels for another 24 h, before harvesting and standard western blotting for the indicated molecules. (**D**) Serially sectioned breast tumor tissues from PyVT mice were stained for the indicated molecules and visualized at ×200 magnification. Black-dotted boxes depict the marginal area invasive to mesenchyme. (**E**) Subconfluent cells were harvested for whole-cell lysates and normalized and processed for standard western blots for the indicated molecules. (**F**) Schematic representation of the promoter regions of human *paxillin* gene with putative c-Jun (thin red vertical lines) or Elk-1 (thin green vertical line) binding sites and the PCR amplification regions (thick horizontal lines) of the chromatin immunoprecipitates. Chromatin immunoprecipitated from cells using normal IgG or anti-c-Jun (left bottom) or anti-Elk-1 (right bottom) antibodies without or with U0126 or YH16899 treatment were processed for PCR using primers for the *PXN* promoter regions or control regions without binding sites. Bp depicts the DNA ladders. Data represent three independent experiments.

Because imaging was commenced 3 h after embedding, we also examined signaling activities at 3 and 24 h after the spheroids were embedded into collagen I gels. Consistent with the laminin-adherent 2D condition (data not shown), c-Src, ERK1/2, and paxillin (but not FAK) activity depended on KRS expression, decreasing with time after the embedding, with the exception of phospho-Tyr118 paxillin, which was found to increase (Figure 2A). Similar decreases in ERK1/2 and paxillin signaling upon KRS suppression were also observed in another colon cancer cell line, SW620, embedded in 3D collagen gels (Figure 2B). The reduced paxillin expression could be mostly due to decreased transcriptional activity in KRS-suppressed cells (Figure 3C), although the half-life of paxillin protein in KRS-suppressed cells might not be shorter than that in KRS-expressing parental cells (Figure S2). KRS suppression also blocked ERK1/2 phosphorylation and paxillin expression in 3D collagen I gels (Figures 2D, 2E), but had no effects on the expression of other focal adhesion molecules such as vinculin, tensin2, and talin (Figure 2F); this suggests a specific effect of KRS suppression on paxillin. KRS suppression decreased the phosphorylations of vinculin Tyr822 and tensin2 Tyr483, but not of talin Ser425 (Figure 2F), presumably describing KRS-dependent, paxillin activity-mediated secondary effects.

### KRS/p67LR/integrin *α*6β1 linkage correlated for ERK1/2 activation

Because KRS-suppression decreased ERK1/2 phosphorylation, we attempted to measure the *in situ* ERK1/2 activity of the cell clones using an ERK biosensor. On laminin-precoated coverglasses in 2% serum-containing media, KRS-positive cells showed greater FRET signals with oscillations, indicative of highly active ERK1/2 activities, as compared with KRS-suppressed cells, which showed a gradual signal decline (Figures 3A, S3, Movies S10 and S11). This KRS-dependent ERK1/2 activation was consistent with the observation that ERK1/2 phosphorylation was increased by KRS overexpression (Figure 2A). The mean FRET signal intensities showed that ERK1/2 activity clearly depended on KRS expression (Figure 3A, bottom).

We then examined how ERK1/2 could be activated through KRS. Since different HCT116 cell clones with various KRS expression levels did not show altered laminin, p67LR, or integrin α6, β1, and β4 expression levels (Figure 1C), because integrins are known to activate ERK1/2 in many cell and tissue systems [11], we determined whether the interaction between KRS, p67LR, and integrin α6β1 could be correlated to ERK1/2 activation, by checking the physical interactions among these proteins. We used myc-KRS immunoprecipitates prepared from cells kept in suspension or reseeded onto laminin-coated dishes in culture media containing 2% FBS to show the complex formation among KRS, p67LR, and integrins α6 and β1 upon cell adhesion, which again could be disrupted by YH16899 treatment (Figure 3B). Interestingly, transient transfection of ERK1 and 2 into KRS-suppressed cells somewhat increased paxillin expression and Tyr118 phosphorylation, in addition to dramatically increasing phospho-ERK1/2 levels (Figure 3C). Using breast tumors from PyVT mouse, we further showed the expression of KRS, p67LR, and integrin α6 in the luminal cells along with the expression of laminin in the basement membrane (Figure 3D). Together these observations suggest the presence of a link between ERK1/2 activity and paxillin expression/phosphorylation in KRS-expressing cells.

The next question we asked was how ERK1/2 activity affected paxillin expression levels. First, we established that c-Jun expression and Ser63 phosphorylation, but not Elk-1, p38, or JNKs expression and phosphorylation, were dependent on KRS expression (Figure 3E). The suppression of Elk-1 did not down-regulate paxillin expression or Tyr118 phosphorylation (data not shown), which may suggest the involvement of c-Jun in KRS-dependent, ERK1/2-mediated paxillin expression. Thus, we then examined whether c-Jun, but not Elk-1, could be linked to paxillin transcription in a KRS-dependent manner, using chromatin immunoprecipitation. Promoter regions that bind c-Jun (Region 1 with a putative binding site at −460 base pairs (bp) out of five binding sites from −447 to −529 bp upstream of the starting point, and nonbinding control region 2) and Elk-1 (a putative binding site at −568 bp for region 3 and nonbinding control region 4) were identified upstream of the human *PXN* (paxillin) gene (Figure 3F, top). Chromatin immunoprecipitated with the anti-c-Jun antibody, but not normal IgG, as shown by a PCR product for the *PXN* promoter region; this product could be abolished by KRS suppression or YH16899 treatment (Figure 3F, left bottom). In contrast, chromatin immunoprecipitated with anti-Elk-1 antibody did not show any amplified PCR product (Figure 3F, right bottom), indicating that ERK1/2-mediated *paxillin* mRNA transcription in KRS-expressing cells might be via c-Jun rather than Elk-1. The interaction between KRS and integrin α6β1 via p67LR may thus be correlated with KRS-mediated ERK1/2 activity and paxillin expression/activity.

### KRS-dependent cell dissemination required ERK1/2 and paxillin phosphorylations

**Figure 4:**
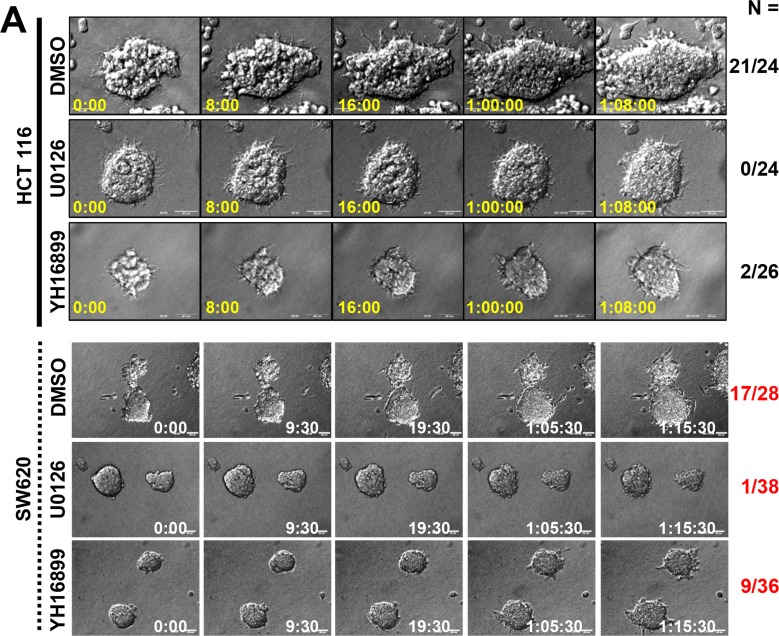

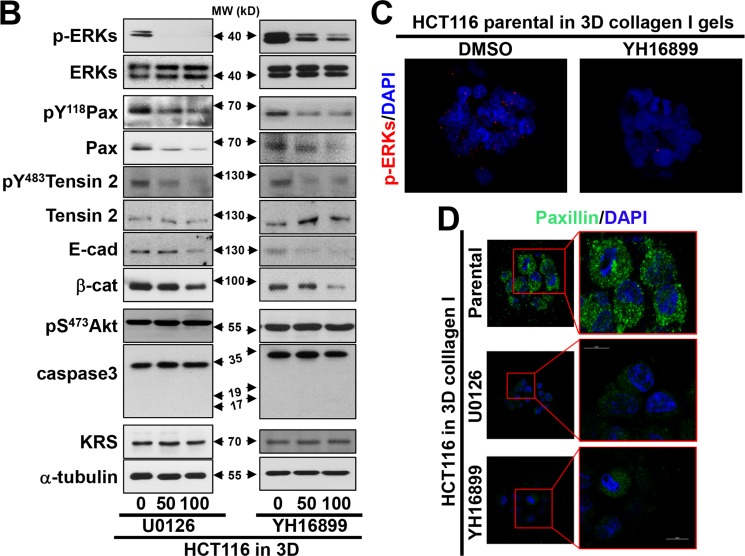

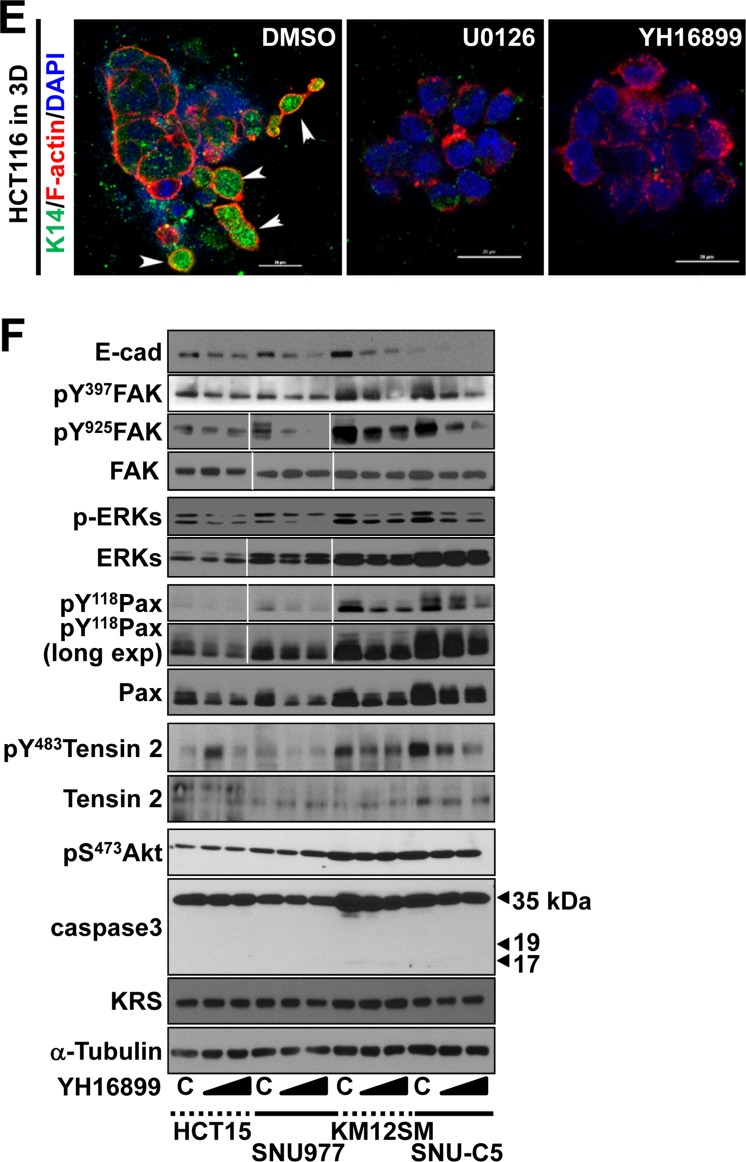
KRS-dependent cell dissemination from spheroids in 3D collagen I gels was blocked by U0126 or YH16899 treatment (**A**) Spheroids of HCT116 or SW620 parental cells embedded in 3D collagen I gels in the presence of DMSO (vehicle control), the ERK1/2 inhibitor U0126 (50 μM), or the KRS inhibitor YH16899 (50 μM) were imaged by time-lapse microscopy for 32 h. The numbers to the right of the figure in the ‘a/b’ format depict disseminative phenotype cases/total experimental cases. See also Movie S12 to S16. (**B**) HCT116 parental spheroids in 3D collagen I gels were treated with U0126 (50 or 100 μM) or YH16899 (50 or 100 μM) for 24 h, and the cells were then processed for standard western blots. (**C**) HCT116 parental spheroids in 3D collagen I gels with DMSO or YH15899 treatment for 24 h were immunostained for pERK1/2 (red) with DAPI (blue) co-staining. (**D** and **E**) Spheroids embedded in 3D collagen I gels for 24 h in the presence of DMSO, U0126, or YH16899 were immunostained for paxillin (green, D) together with DAPI (blue, D), or the epithelial marker K14 (green, E) together with F-actin (red) and DAPI (blue) (**E**). Right panels are enlarged from the red boxes in the corresponding left panels (**D**). Arrow heads depict cells presumably disseminating with K14 expression (**E**). (**F**) Subconfluent colon cancer cells were treated with DMSO (**C**) or YH16899 (increasing doses from 50 to 100 μM) for 24 h, prior to harvesting of whole-cell lysates and immunoblotting for the indicated molecules. Data represent three independent experiments.

Next we explored whether KRS-dependent ERK1/2 activation was critical for dissemination. KRS-expressing parental HCT116 spheroids exhibited dissemination of single or small group of cells (Movie S12), which could be blocked by the MEK inhibitor, U0126 (leading to ERK1/2 inhibition, Movie S13) or YH16899 (Movie S14) (Figure 4A). Treating SW620 cells with U1026 or YH16899 also blocked dissemination (Figure 4A, Movies S7, S15, and S16). Each inhibitor decreased ERK1/2 phosphorylation, paxillin expression and phosphorylation, and did not activate caspase 3; it also caused a loss of epithelial markers (Figure 4B). These inhibitor-mediated effects were very similar to the effects observed by KRS suppression (Figures 1C, 1D, 2A). YH16899 treatment, by comparison, decreased phospho-ERK1/2 in 3D collagen I gels (Figure 4C). Both inhibitors were also able to decrease paxillin protein levels in KRS-suppressed cells in 3D collagen I gels (Figure 4D). Interestingly, U0126 or YH16899 treatment also decreased K14 protein at the disseminative margins (Figure 4E). This was verified in other colon cancer cells showing decreases in ERK1/2 and paxillin expression and paxillin activity following YH16899 treatment (Figure 4F). In the case of FAK phosphorylation, cells with lower FAK Tyr397 phosphorylation levels were not significantly sensitive to YH16899, whereas cells with higher levels were sensitive (Figure 4F).

### KRS-suppression-inhibited dissemination was recovered by ERK1 and/or paxillin expression

**Figure 5:**
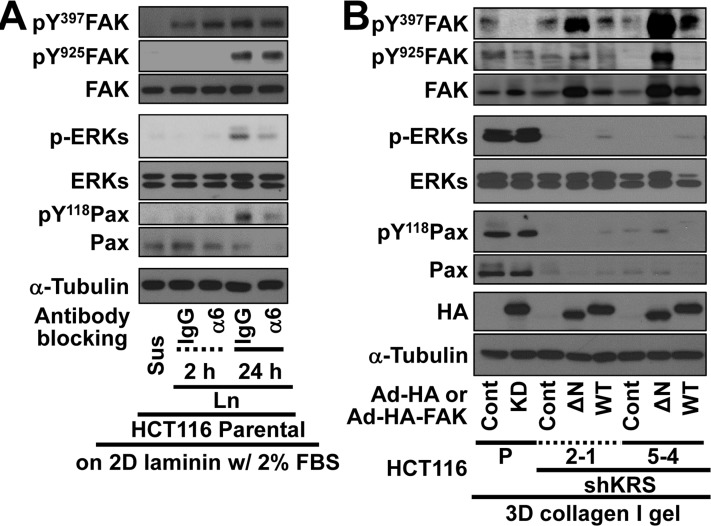

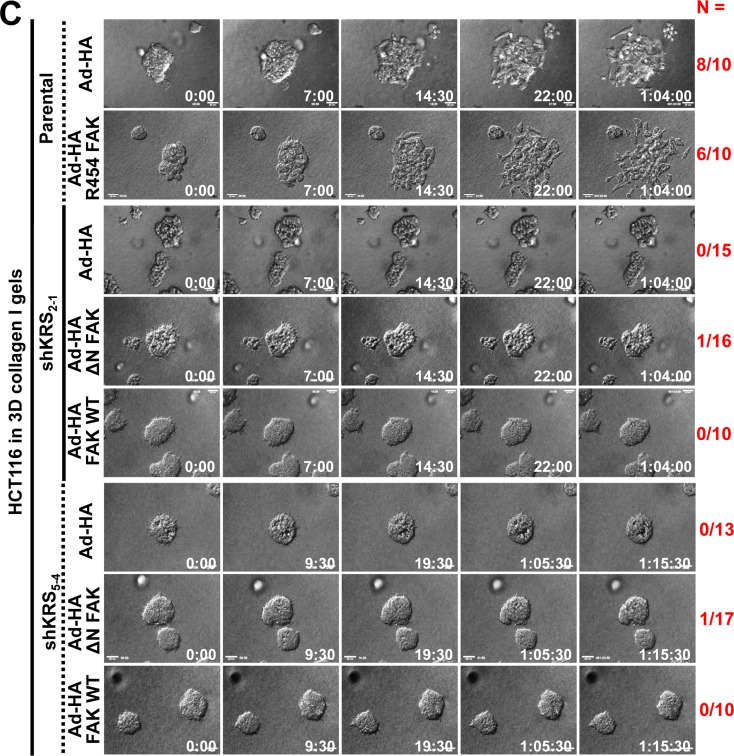

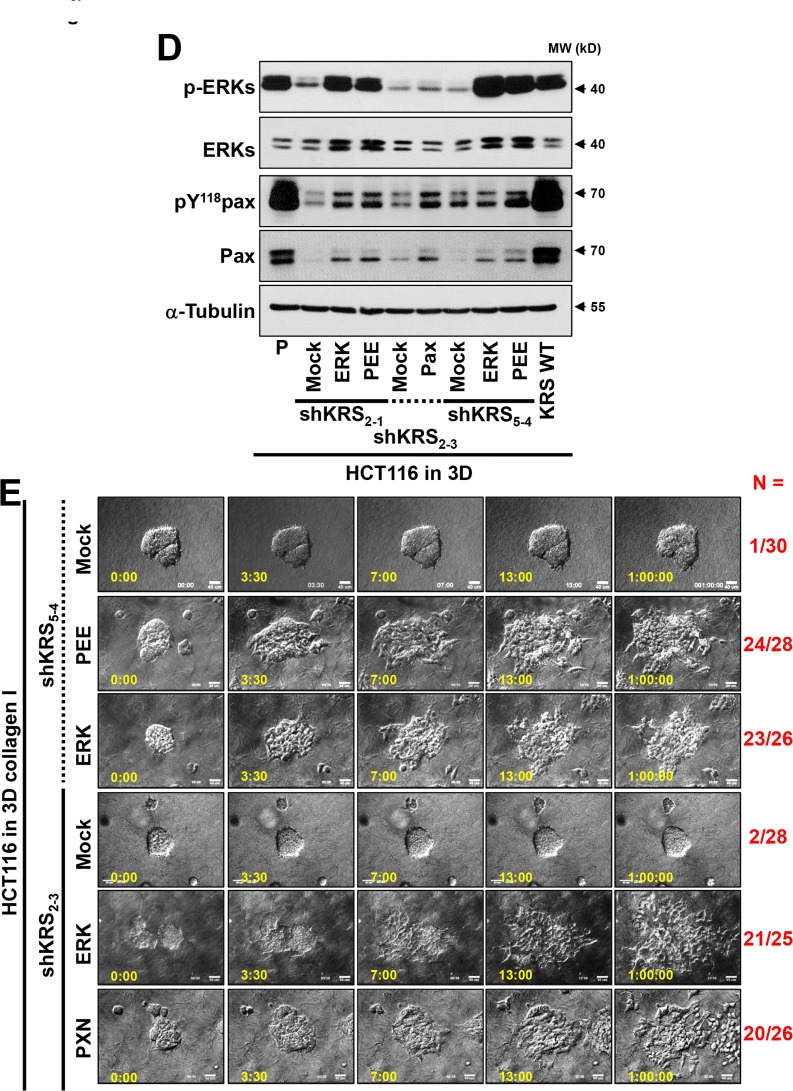
Blockade of dissemination of KRS-suppressed cells was relieved by ERK1/2 and/or paxillin expression (**A**) HCT116 parental cells were suspended in serum-free BSA containing media, preincubated with normal IgG or functional anti-human integrin α6 blocking antibody (20 μg/ml) for 30 min, and then kept in suspension or reseeded onto laminin-precoated culture dishes with 2% serum-containing media for 2 or 24 h. Whole-cell lysates were immunoblotted for the indicated molecules. (**B** and **C**) Cells were transduced with adenovirus (Ad-HA) for R454 (kinase dead) FAK, ΔN(1-100) FAK, or FAK WT for 12 h. The cells were embedded in 3D collagen I gels and, 3 h later, the cell lysates were prepared for immunoblotting (**B**) or time-lapse imaging was performed for a further 28 h (1:04:00, C). The numbers to the right of the figure in the ‘a/b’ format depict disseminative phenotype cases/total experimental cases. See also Movie S17 to 24. In anti-HA antibody blot, the exogenous FAK showed two different sizes due to WT and its N-terminal deletion, but FAK phosphorylation blots appeared to be single band due to robust phosphorylations mediated by the N-terminal deletion mutant FAK [29]. (**D** and **E**) KRS-expressing cells and KRS-suppressed HCT116 cell spheroids (2-1, 2-3 and 5-4 clones) with stable transfection of ERK1/2 (ERK), paxillin (PXN), or ERKs/paxillin (PEE) were embedded in 3D collagen I gels for 24 h, prior to harvesting whole-cell lysates for immunoblottings (**D**). Alternatively, the spheroids were embedded for 3 h and then time-lapse imaged for another 24 h (**E**). See also Movie S25 to 30. Data represent three independent experiments.

We explored whether ERK1/2 activity in KRS-expressing cells depended on FAK. Because KRS-dependent ERK1/2 activity appeared to occur via the complex formation of KRS, p67LR, and integrin α6β1, we examined how functional-blocking of integrin α6 affected the activities of FAK and ERK1/2. When HCT116 parental cells were preincubated with anti-human integrin α6 neutralizing antibodies, we observed a decrease in ERK1/2 activity and paxillin expression/Tyr118 phosphorylation, in a manner dependent on cell-laminin adhesion in 2% FBS-containing media; FAK phosphorylation, however, did not decrease under these conditions (Figure 5A). We then examined FAK, ERK1/2, and paxillin expressions and activities using HCT116 clone spheroids in 3D collagen I gels following FAK adenovirus infections. We found that the expression of kinase-dead (R454) FAK in KRS-expressing cells led to an expected decrease in FAK phosphorylation, but had no effects on ERK1/2 activity or paxillin expression and Tyr118 phosphorylation (Figure 5B, lanes 1 and 2). The expression of active FAK [N-terminal deleted FAK [12]] or wild-type FAK in KRS-suppressed cells increased FAK phosphorylation, but also did not alter ERK1/2 activity or paxillin expression and Tyr118 phosphorylation (Figure 5B, lanes 3 to 8). This absence of a link between FAK and ERKs/paxillin activity and expression downstream of KRS was also observed for the dissemination from spheroids; although R454 FAK expression in KRS-expressing cells did not significantly block dissemination, and the expression of active or wildtype FAK in KRS-suppressed cells did not cause dissemination (Figures 5C, Movies S17 to S24). These experiments suggested that KRS-dependent dissemination might involve FAK-independent ERK1/2 activity.

We next examined whether this absence of dissemination of KRS-suppressed cells could be recovered by the overexpression of ERK1 or paxillin alone or together. Stable transfection of ERK1/2 resulted in ERK1/2 activation, and a slight induction of paxillin expression and Tyr118 phosphorylation (Figure 5D), as shown also in Figure 3C. In addition, the stable transfection of paxillin alone or together with ERK1 resulted in moderately increased paxillin Tyr118 phosphorylation (Figure 5D). Using time-lapse microscopy, we show that the stable transfection of ERK1 and/or paxillin recovered the dissemination of KRS-suppressed cell clones from spheroids (Figure 5E, Movies S25 to S30).

Meanwhile, we tested whether the lack of dissemination in KRS-suppressed cells might be attributed to a lack of protease activity. MT1-MMP is a dominant proteolytic effector during cell trafficking in 3D collagen I gels [13]. We found that MT1-MMP-transfection in KRS-suppressed cells did not cause dissemination, although MT1-MMP transfected cells were disseminated in parental HCT116 spheroids (Figure S4, upper panels). However, treatment with U0126 or YH16899 blocked the dissemination of parental cells even after MT1-MMP transfection (Figure S4, lower panels). Thus, the dissemination of KRS-expressing cells might need more than the MT1-MMP-based proteolytic activity.

### KRS appeared to have pro-metastatic roles at the invasive margins of KRS^−/+^ mouse breast tumor and human colon tumor tissues

**Figure 6:**
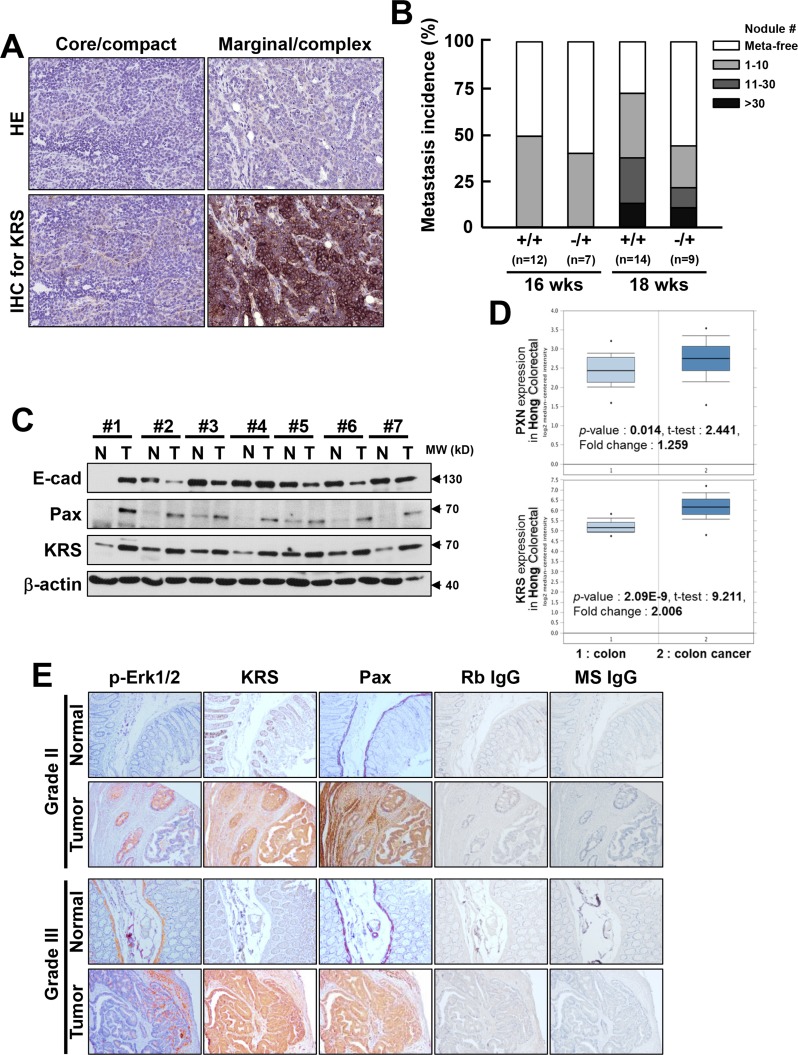

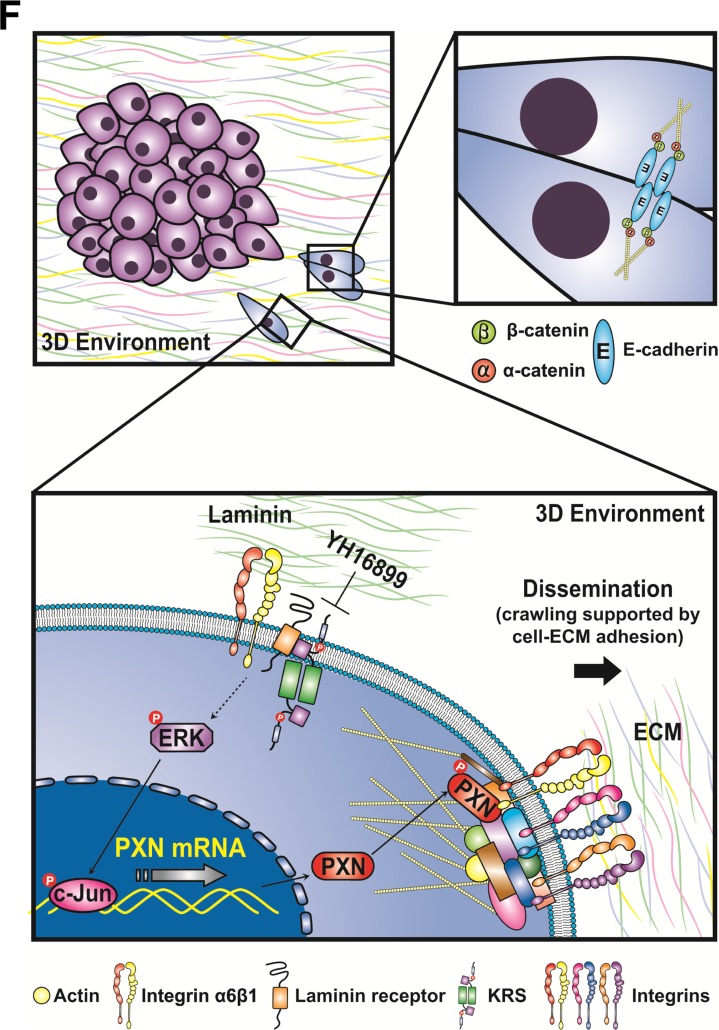
Relevance of KRS in tumor metastasis in KRS-/+ PyVT mouse and colon cancer patient tissues (**A**) Breast tumor sections of the compact core or marginal region in PyVT mice were stained with hematoxylin-eosin (HE) or immunostained for KRS (KRS IHC). (**B**) Metastatic lung nodules were counted in the PyVT mice with the indicated *kr*s genotypes (WT or KRS^−/+^ heterozygosity) at 16 or 18 weeks of age. (**C**) Normal (N) or tumor (T) colon tissues from different clinical colon cancer patients were harvested for whole-tissue extracts at 4°C, prior to standard western blotting for the indicated molecules. Data shown represent three independent experiments. See also Figure S5. (**D**) Data from Oncomine online cancer genomics data analysis tools showed the significant overexpression of KRS and paxillin in a positively-correlated manner in colon carcinoma, as compared with normal colon tissues. (**E**) Serial sections (6 μm) from normal or tumor tissues from grade II or grade III colon cancer patients were processed for immunohistochemistry using the indicated antibodies in addition to controls using normal rabbit (Rb) or mouse (MS) IgG. (**F**) Scheme to represent KRS-mediated cell dissemination from spheroids in 3D collagen I gels via the regulation of cell-cell and cell-ECM adhesion. KRS expression maintains E-cadherin-based cell-cell junctions. Laminin expressed in an autocrine manner binds to p67LR, which forms a complex with KRS and integrin α6β1, leading to ERK1/2 activation, which in turn causes paxillin expression via c-Jun-dependent transcriptional activation and Tyr118 phosphorylation. Activated paxillin can mediate cell-ECM adhesion-related signaling and/or contractility force generation to facilitate dissemination or crawling out.

Next, we explored the roles of KRS in lung metastasis of breast tumors using KRS-heterozygous MMTV-PyVT mice. A genetrap cassette was inserted into intron 13 to disrupt KRS gene expression (Figure S5A, S5B). A comparison of KRS immunoblots in various organs from wild-type and heterozygous mice demonstrated the overall reduction of KRS (Figure S5C). When breast tumor sections in PyVT mice were analyzed, the compact core region of the tumor section showed very weak KRS immunostaining, whereas marginal regions with highly complex lumen structures showed strong KRS-staining (*n* = 4, Figure 6A). KRS-heterozygous PyVT mice showed a lower incidence of metastasis than KRS wild-type mice, as determined by counting metastatic lung nodules in the PyVT mice at 16 or 18 weeks (Figure 6B). Therefore, this KRS^−/+^ mouse model suggests that KRS might play roles at the invasive margins of metastatic tumor.

Following on from this, we lastly examined the expressions of KRS, pERK1/2, and paxillin in clinical colon cancer tissues. Compared with normal colon tissues, tumor tissues showed higher KRS and paxillin expression but lower E-cadherin expression (Figure 6C), suggesting a positive correlation between KRS and paxillin (7/7) but a negative correlation between KRS and E-cadherin (4/7). Phospho-ERK1/2 was not easily detected in either normal or tumor colon tissue extracts (data not shown), presumably because the extracts were from populations mixed with different cell types around the tumor lesions. Further, the Oncomine database (www.oncomine.org) showed a positive correlation between the significant overexpression of KRS and paxillin in colon carcinoma samples (Figure 6D). When using serial sections of normal and tumor colon tissues, tumor tissues identified as grade II or III clearly showed marginal and local staining for pERK1/2, whereas KRS and paxillin were observed not only in local but also in core areas (Figure 6E).

## DISCUSSION

The observations in this study made using diverse systems *in vitro* cells embedded in 3D collagen I gels, KRS^−/+^ heterozygous mice, and clinical tumor tissues, support the notion that KRS functions together with p67LR/integrin α6β1 receptors to mediate cell dissemination from (tumor) cell masses via the laminin adhesion-dependent regulation of ERK1/2 and paxillin expression and activity. In addition to KRS-dependent E-cadherin expression, ERK1/2 activity and paxillin expression and activity following the formation of a complex between KRS, p67LR, and integrin α6β1 are thus important for the dissemination of single and/or small-groups of cells from colon cancer spheroids embedded in 3D collagen I gels (Figure 6F). This KRS/ERKs/paxillin signaling axis was further implicated in marginal invasiveness and metastasis observed in mouse animal models and in clinical colon tissue models. This study also provides a platform for KRS-dependent cell dissemination from tumor masses in 3D collagen I gels, which may be useful to screen therapeutic reagents against KRS-dependent cancer metastasis.

Here we show that KRS is required for dissemination of single or small-groups of cells from colon cancer spheroids in 3D collagen I gels, where E-cadherin and K14 were maintained at the cell-cell junctions; this suggests a new mode of KRS-dependent dissemination in cells that retain epithelial markers at the junctions. Such a role for KRS has never been explored. This new dissemination mode is possibly different from the collective cell migration/invasion theory [14, 15], as cells in collective migration and invasion may not always involve cell-substrate adhesion but cell-cell adhesion. Colon cancer dissemination is suggested to undergo EMT-like de-differentiation at the invasive front to detach and migrate; the dissemination occurs through inter-switching between reversion of single cells to epithelial plates (via the MET process) and then back to single cells (via EMT again) [16]. HCT116 cells express E-cadherin in high amounts and are categorized as the most epithelial-like cell line in the EMT score among diverse tumors [17]. Presumably, E-cadherin in KRS-positive colon cancer cells may not have exceptional intercellular adhesiveness and the cell-cell adhesions in these cells may be easily overcome in favor of cell-substrate adhesion to execute a transient EMT-like process.

KRS regulates cell-substrate adhesion during dissemination. KRS is phosphorylated at Ser207 by ERK1/2 for nuclear translocation to regulate microphthalmia-associated transcription factor (MITF)-dependent gene expression in immunologically stimulated mast cells [5]. Laminin-induced p38MAPK-dependent Thr52 phosphorylation in KRS causes to translocate from the cytosolic multi-tRNA synthetase complex to the plasma membrane, where it protects p67LR from ubiquitination-mediated degradation [6]. An interaction between KRS and p67LR has been observed in lung metastases of subcutaneously-injected mouse breast carcinoma 4T1 cells with KRS overexpression [7]. In this study, we observed that the KRS/p67LR complex also includes integrin α6β1, which allows KRS-expressing cells to transduce intracellular signals under extracellular laminin-stimulated conditions. We observed that KRS expression activates ERK1/2 for paxillin expression and activity. This, in turn, leads to secondary phosphorylation events in other focal adhesion molecules during dissemination, including vinculin and tensin2, which are important for myosin contractility-dependent adhesion strength and traction force of mouse embryonic fibroblasts [18] as well as contractility of human foreskin fibroblasts in 3D collagen gels [19]. Dissemination thus requires cell-substrate adhesion signaling that is supported by paxillin Tyr118 phosphorylation to generate a sufficient contractility force to crawl out. Consistently, epithelial scattering is driven by changes in cellular contractility alone [20].

Here, KRS-dependent ERKs activity induced paxillin and its phosphorylation. Paxillin localizes to focal adhesions [21]. Although paxillin is phosphorylated by active FAK [22], this study has revealed that paxillin expression is regulated by ERK1/2 and c-Jun activities. Activated ERK1/2 correlates with total paxillin expression and Ser178 phosphorylation but not with Tyr118 phosphorylation during EGF/TGFβ1-mediated hepatocyte migration [23]. The 3D culture using collagen I gels supplied with 10% FBS-containing culture media in this study efficiently showed laminin expression independent of KRS suppression, suggesting that laminin is available during the experiments in the 3D collagen I gels. Thus, it is likely that KRS may mediate ERK1/2 activation for pro-metastatic roles following specific adhesion-mediated signals from the extracellular microenvironment, including laminin.

Here, KRS-dependent dissemination was shown to require ERK1/2, but not FAK, for paxillin expression. KRS-overexpressing HCT116 cells showed greater FAK activity than parental HCT116 cells, as shown in previous studies of KRS-overexpressing lung adenocarcinoma A549 cells [6] or lung squamous carcinoma NCI-H226 cells [7], which activate FAK upon being reseeded on laminin. However, here KRS-suppressed colon carcinoma HCT116 cells in 3D collagen I gel conditions still showed FAK activity comparable to that in KRS-expressing cells, and FAK activity appeared to not have a role in the 3D dissemination of HCT116 cells. These observations may suggest that a correlation between KRS and FAK was valid only when KRS was expressed in high enough concentrations, and that KRS-dependent ERKs activation occurred here in FAK-independent manners [24, 25]. Although this study has focused on non-canonical role of KRS on membranes in invasive migration for cancer dissemination, it may not be ruled out that KRS in cytosol and/or nucleus might contribute to the KRS-dependent metastasis.

Taken together, the linkage from the KRS/p67LR/integrin α6β1 complex to ERKs/c-Jun activity for paxillin expression/phosphorylation, in addition to maintaining the E-cadherin-based cell-cell contacts, could thus be the underlying mechanism for KRS as a pro-metastatic molecule. KRS may therefore represent a promising therapeutic target to address (colon) cancer metastasis.

## MATERIALS AND METHODS

### Cells

HCT116 and SW620 colon cancer (ATCC, Manassas, VA) and other colon cancer cells (Korean Cell Bank, Seoul National Univ.) were stably transfected with shRNA against KRS (transcript 1, NM_001130089, lysyl-tRNA synthetase MISSION^®^ shRNA Plasmid DNA, Sigma-Aldrich, St Louis, MO) using Truefect (United Biosystems, Herndon, VA) before antibiotic selections. The target sequences of shKRS and the names of the KRS-suppressed cell clones are shown in Table 1. HCT116 cells overexpressing KRS were established via the stable transfection of a myc-tagged KRS plasmid [6]. ERK1, ERK2, and paxillin were also stably transfected, either separately or together, into KRS-suppressed cell clones. Stable myc-tagged KRS and different stable shKRS-transfected clones were established by using 250 μg/ml G418 or 0.50 μg/ml puromycin (AG Scientific, Inc., San Diego, CA). The cells were maintained in RPMI-1640 (WelGene, Daegu, Korea) containing 10% FBS and antibiotics (Invitrogen, Grand Island, NY).

**Table 1:** Stable HCT116 cell clones with suppressed KRS expression levels after transfections of shRNA against KRS

shKRS	Target sequence in KRS (NM-001130089.1)	Stable clones
shKRS0	CCGG^1581^CCMGAAGTGACTTGCATCAA^1601^CTCGAGTTGATGCAAGTCACTTCCAGG TTTTTG (exon 12)	0-1, 0-2, 3
shKRS-1	CCG^437^GCGTGGACCCAAATCAATACTAC^459^TCGAGTA GTATTGATTTGGGTCCACGTTTTTG (exon 3-4)	
shKRS-2	CCG^911^GCCAGAGATACTTGGACTTGATC^933^TCGAGATCAAGTCCAAGTATCTCTGG TTTTTG (exon 7)	2-1, 2-2, 2-3
shLRS5	CCGG^1071^GCCTTTCATCACTTATCACAAC^1092^TCGAGTTGTGATAAGTGATGAAAGGCTTTTTG (exon 12)	5-4, 5-10

### Spheroid formation and embedding into 3D collagen I gels

Cells with modulated KRS expression levels were processed for spheroid formation using Perfecta3D^®^ 96-well hanging-drop plates (3D Biomatrix, Ann Arbor, MI). Spheroids were selected to be less than 70 μm in size using a cell strainer sieve (SPL Life Science Co., Pocheon-si, Korea) prior to being embedded into 3D collagen I gels (BD Biosciences, San Jose, CA), as explained in a previous study [26].

### Time-lapse imaging of cells in 3D ECM gels

Three hours after spheroids were embedded in 3D collagen I gels, time-lapse images were collected for the indicated periods using IX81-ZDC microscope (Olympus). The microscope was equipped with a 10-well Chamlide Incubator system (Live Cell Instrument, Seoul, Korea), and an environmental chamber maintained constant conditions of 37°C, 5% CO_2_, and 95% humidity. U0126 (50 μM) or YH16899 [50 μM, [7]] were mixed during embedding of the cells into 3D collagen I gels. Scale bars depict 40 μm.

### Extract preparation and western blots

Cells within collagen I gels were washed with ice-cold PBS and then homogenized with truncated pipette tips (3 times for 20 min each on ice) in modified RIPA buffer (50 mM Tris-HCl, 150 mM NaCl, 1% NP-40, and 0.25% sodium deoxycholate) with a protease inhibitor cocktail (GenDepot, Barker, TX), as explained in a previous study [26]. The primary antibodies used were as follows: α-tubulin, α-SMA, talin, and vimentin (Sigma-Aldrich); pY^416^c-Src, ERK1/2, phospho-ERK1/2, FLAG, pS^473^Akt, pS^425^talin, caspase 3, and Akt (Cell Signaling Technology, Danvers, MA); paxillin, N-cadherin, and FAK (BD Biosciences); pY^397^FAK, p67LR, laminin, and Twist1 (Abcam, Cambridge, UK); c-Src, pY^118^paxillin, pY^577^FAK, pY^861^FAK, pY^925^FAK, Snail1, E-cadherin, β-catenin, and Slug (Santa Cruz Biotechnology, Dallas, TX); ZO1 (Zymed Laboratories, Camarillo, CA); vinculin, integrin α6, β1, and β4 (Millipore, Billerica, MA); fibronectin (DAKO, Carpinteria, CA); and KRS (Atlas Antibodies, Stockholm, Sweden).

### RT-PCR

Total RNA was extracted from cells, using TRIzol (Invitrogen) according to the manufacturer's protocol. One microgram of total RNA was reverse transcribed using the amfiRivert Platinum cDNA Synthesis master mix (GenDepot). Primers were designed using Primer3 software as follows: human *paxillin (PXN)* mRNA, forward 5′-GAAATCAGCTGAGCCTTCAC-3′ and reverse 5′-TTAGGCTTCTCTTTCGTCAGG-3′, human *KRS* (*KARS*) mRNA, forward 5′- CAATGCCCATGCCCCAGCCA-3′ and reverse 5′- ACCCCACCCTTCCGGCGAAT-3′, and human β*-actin* (*ACTB*) mRNA, forward 5′-TGACGGGGTCACCCACACTGTGCCCATCTA-3′ and reverse 5′-CTAGAAGCATTTGCGGTGGACGACGGAGGG-3′.

### Normal 2D culture or replating on 2D ECM layer

Cells were kept in suspension or replated on ECM (10 μg/ml, laminin or fibronectin, BD Biosciences)-precoated dishes or coverglasses in the absence (in the case of fibronectin) or presence of low serum (2%, in the case of laminin) for 1 h before being analyzed for cell-ECM adhesion signaling by standard western blotting or for the formation of focal adhesions by indirect immunofluorescence, as described previously [27]. Function blocking antibodies against human integrin α6 (Millipore, 20 μg/ml) were preincubated with cells by rocking at 37°C for 1 h prior to replating. Pharmacological inhibitors were added to the culture media for 24 h or to replating media at the reseeding time.

### Time-lapse FRET analysis for ERK1/2 activity

HCT116 cells were cultured on a laminin-coated (5 μg/ml), 35-mm, glass-base dish and were transfected with FRET-based indicators [28] using the Lipofectamine 2000 (Life Technologies, Grand Island, NY), according to the manufacturer's instructions.

### Coimmunoprecipitations

Whole-cell extracts prepared from cells in standard media containing 10% FBS or from cells kept in suspension or reseeded onto laminin (10 μg/ml) in media containing 2% FBS for 2 h were immunoprecipitated overnight using an anti-myc tagged antibody-coated agarose beads (Sigma-Aldrich, St Louis, MO). The immunoprecipitated proteins were boiled in 2× SDS-PAGE sample buffer before processing using standard western blot techniques.

### Indirect immunofluorescence

Cells reseeded in 2D normal culture media or onto ECM-precoated glass coverslips or embedded in 3D collagen I gels within PDMS were immunostained using antibodies against E-cadherin, snail1, pERK, K14, or paxillin in addition to DAPI staining for the nucleus. Immunofluorescence images were acquired on a fluorescence microscope (BX51TR, Olympus, Tokyo, Japan) or a confocal laser scanning microscope with a Nikon Plan-Apochromat 60×/1.4 N.A. oil objective (Nikon eclipse Ti microscope, Nikon, Tokyo, Japan), and then analyzed using the IMARIS imaging software (Bitplane AG, Zurich, Switzerland), as explained in a previous study [26].

### Chromatin immunoprecipitation (ChIP)

ChIP assays were performed using the ChIP-IT Express kit (Active Motif, Carlsbad, CA), following the manufacturer's protocols.

### Analyses of murine or human tissues

All procedures for animal and human tissues were performed in accordance with the procedures of the Seoul National University Laboratory Animal Maintenance Manual and Institutional Review Board (IRB) agreement approved by the Institute of Laboratory Animal Resources Seoul National University (ILARSNU) and SNUIRB, respectively. Human colon tissues were obtained with informed content from patients who received surgery at Samsung Medical Center (Sungkyunkwan University School of Medicine, Seoul, Korea) or National Biobank of Korea-Pusan National University Hospital (PNUH) in accordance with IRB agreements. Whole-tissue extracts were prepared with modified RIPA buffer [27] using a Bullet Blender BBX24 surrounded by dry ice and containing 1.0 mm zirconium silicate beads (Next Advance, Inc. Averill Park, NY). Protein lysates were then subjected to standard western blot analyses for the molecules, as indicated in each figure.

### Analyses of KRS heterozygote mouse

For the generation of KRS heterozygote animals, KRS genetrap ES cells (AJ0130) were purchased from the MMRRC (https://www.mmrrc.org/index.php) and used to generate a chimeric mouse at the UC San Diego Moores Cancer Center transgenic mouse core facility (San Diego, CA). The disruption of the gene via the insertion of the genetrap cassette into intron 13 was confirmed by genomic PCR using primers flanking the breakpoint (forward: 5′- TGG GTT TCA TCC TGA GGT CT - 3′; reverse: 5′- GCT TTC TTT CCC AGG TCC TC - 3′; genetrap reverse: 5′-TGT CCT CCA GTC TCC TCC AC - 3′). No viable homozygous pups were born, nor were any viable embryos observed (up to embryonic day 9.5). MMTV-PyVT mice (The Jackson Laboratory, Bar Harbor, MA) were crossed to KRS heterozygotes to generate the MMTV-PyVT; KRS^+/+^ and MMTV-PyVT; KARS^+/GT^ mice. Female mice were sacrificed at 16 and 18 weeks. After the lungs were dissected out and fixed, each lobule was carefully examined under the microscope to count the total number of metastatic nodules. A white round bump of over 100 μm on the lung surface was counted as a nodule.

### Immunohistochemistry

Dissected tumor tissues were fixed and stored in 10% neutral-buffered formalin. The tissues were processed, paraffin embedded, and sectioned at 6-μm thickness. Immunohistochemistry of serial sections of paired normal or tumor colon tissues was performed using normal rabbit IgG, normal mouse IgG, phospho-ERKs (Cell Signaling Technology), KRS (Atlas Antibodies, Stockholm, Sweden), and paxillin (BD Biosciences, San Jose, CA), as explained in a previous study [7].

### Statistical methods

Student's *t*-test was performed for statistical comparisons of mean values. A *p*-value less than 0.05 was considered statistically significant.

Supplemental information accompanies the paper on the journal website.

## SUPPLEMENTARY MATERIAL FIGURES AND MOVIES































































## References

[1] Thiery JP, Lim CT (2013). Tumor Dissemination: An EMT Affair. Cancer Cell.

[2] Meng F, Wu G (2012). The rejuvenated scenario of epithelial-mesenchymal transition (EMT) and cancer metastasis. Cancer Metastasis Rev.

[3] Kim S, You S, Hwang D (2011). Aminoacyl-tRNA synthetases and tumorigenesis: more than housekeeping. Nat Rev Cancer.

[4] Park SG, Kim HJ, Min YH, Choi EC, Shin YK, Park BJ, Lee SW, Kim S (2005). Human lysyl-tRNA synthetase is secreted to trigger proinflammatory response. Proc Natl Acad Sci U S A.

[5] Yannay-Cohen N, Carmi-Levy I, Kay G, Yang CM, Han JM, Kemeny DM, Kim S, Nechushtan H, Razin E (2009). LysRS serves as a key signaling molecule in the immune response by regulating gene expression. Mol Cell.

[6] Kim DG, Choi JW, Lee JY, Kim H, Oh YS, Lee JW, Tak YK, Song JM, Razin E, Yun SH, Kim S (2012). Interaction of two translational components, lysyl-tRNA synthetase and p40/37LRP, in plasma membrane promotes laminin-dependent cell migration. FASEB J.

[7] Kim DG, Lee JY, Kwon NH, Fang P, Zhang Q, Wang J, Young NL, Guo M, Cho HY, Mushtaq AU, Jeon YH, Choi JW, Han JM, Kang HW, Joo JE, Hur Y (2014). Chemical inhibition of prometastatic lysyl-tRNA synthetase-laminin receptor interaction. Nat Chem Biol.

[8] Canfield SM, Khakoo AY (1999). The nonintegrin laminin binding protein (p67 LBP) is expressed on a subset of activated human T lymphocytes and, together with the integrin very late activation antigen-6, mediates avid cellular adherence to laminin. J Immunol.

[9] Ardini E, Tagliabue E, Magnifico A, Buto S, Castronovo V, Colnaghi MI, Menard S (1997). Co-regulation and physical association of the 67-kDa monomeric laminin receptor and the α6β4 integrin. J Biol Chem.

[10] Berno V, Porrini D, Castiglioni F, Campiglio M, Casalini P, Pupa SM, Balsari A, Menard S, Tagliabue E (2005). The 67 kDa laminin receptor increases tumor aggressiveness by remodeling laminin-1. Endocr Relat Cancer.

[11] Lee JW, Juliano R (2004). Mitogenic signal transduction by integrin- and growth factor receptor-mediated pathways. Mol Cells.

[12] Jung O, Choi S, Jang SB, Lee SA, Lim ST, Choi YJ, Kim HJ, Kim DH, Kwak TK, Kim H, Kang M, Lee MS, Park SY, Ryu J, Jeong D, Cheong HK (2012). Tetraspan TM4SF5-dependent direct activation of FAK and metastatic potential of hepatocarcinoma cells. J Cell Sci.

[13] Rowe RG, Weiss SJ (2009). Navigating ECM barriers at the invasive front: the cancer cell-stroma interface. Annu Rev Cell Dev Biol.

[14] Thiery JP (2009). Metastasis: Alone or Together?. Current Biology.

[15] Friedl P, Locker J, Sahai E, Segall JE (2012). Classifying collective cancer cell invasion. Nat Cell Biol.

[16] Chua KN, Poon KL, Lim J, Sim WJ, Huang RY, Thiery JP (2011). Target cell movement in tumor and cardiovascular diseases based on the epithelial-mesenchymal transition concept. Adv Drug Deliv Rev.

[17] Huang RY, Wong MK, Tan TZ, Kuay KT, Ng AH, Chung VY, Chu YS, Matsumura N, Lai HC, Lee YF, Sim WJ, Chai C, Pietschmann E, Mori S, Low JJ, Choolani M (2013). An EMT spectrum defines an anoikis-resistant and spheroidogenic intermediate mesenchymal state that is sensitive to e-cadherin restoration by a src-kinase inhibitor, saracatinib (AZD0530). Cell Death Dis.

[18] Dumbauld DW, Lee TT, Singh A, Scrimgeour J, Gersbach CA, Zamir EA, Fu J, Chen CS, Curtis JE, Craig SW, García AJ (2013). How vinculin regulates force transmission. Proceedings of the National Academy of Sciences.

[19] Clark K, Howe JD, Pullar CE, Green JA, Artym VV, Yamada KM, Critchley DR (2010). Tensin 2 modulates cell contractility in 3D collagen gels through the RhoGAP DLC1. J Cell Biochem.

[20] Hoj JP, Davis JA, Fullmer KE, Morrell DJ, Saguibo NE, Schuler JT, Tuttle KJ, Hansen MDH (2014). Cellular contractility changes are sufficient to drive epithelial scattering. Exp Cell Res.

[21] Katoh K, Masuda M, Kano Y, Jinguji Y, Fujiwara K (1995). Focal adhesion proteins associated with apical stress fibers of human fibroblasts. Cell Motility & the Cytoskeleton.

[22] Bellis SL, Miller JT, Turner CE (1995). Characterization of tyrosine phosphorylation of paxillin *in vitro* by focal adhesion kinase. J Biol Chem.

[23] Guller MC, Andre J, Legrand A, Setterblad N, Mauviel A, Verrecchia F, Daniel F, Bernuau D (2009). c-Fos accelerates hepatocyte conversion to a fibroblastoid phenotype through ERK-mediated upregulation of paxillin-Serine178 phosphorylation. Mol Carcinog.

[24] Wary KK, Mariotti A, Zurzolo C, Giancotti FG (1998). A requirement for caveolin-1 and associated kinase Fyn in integrin signaling and anchorage-dependent cell growth. Cell.

[25] Lu KK, Armstrong SE, Ginnan R, Singer HA (2005). Adhesion-dependent activation of CaMKII and regulation of ERK activation in vascular smooth muscle. Am J Physiol Cell Physiol.

[26] Lee MS, Kim S, Kim BG, Won C, Nam SH, Kang S, Kim HJ, Kang M, Ryu J, Song HE, Lee D, Ye SK, Jeon NL, Kim TY, Cho NH, Lee JW (2014). Snail1 induced in breast cancer cells in 3D collagen I gel environment suppresses cortactin and impairs effective invadopodia formation. Biochim Biophys Acta.

[27] Lee SA, Kim YM, Kwak TK, Kim HJ, Kim S, Kim SH, Park KH, Cho M, Lee JW (2009). The extracellular loop 2 of TM4SF5 inhibits integrin α2 on hepatocytes under collagen type I environment. Carcinogenesis.

[28] Harvey CD, Ehrhardt AG, Cellurale C, Zhong H, Yasuda R, Davis RJ, Svoboda K (2008). A genetically encoded fluorescent sensor of ERK activity. Proc Natl Acad Sci USA.

[29] Lim S-T, Chen XL, Lim Y, Hanson DA, Vo T-T, Howerton K, Larocque N, Fisher SJ, Schlaepfer DD, Ilic D (2008). Nuclear FAK Promotes Cell Proliferation and Survival through FERM-Enhanced p53 Degradation. Molecular Cell.

