# Erratum: Noncanonical roles of membranous lysyl-tRNA synthetase in transducing cell-substrate signaling for invasive dissemination of colon cancer spheroids in 3D collagen I gels

**DOI:** 10.18632/oncotarget.7117

**Published:** 2016-02-01

**Authors:** Seo Hee Nam, Doyeun Kim, Mi-Sook Lee, Doohyung Lee, Tae Kyoung Kwak, Minkyung Kang, Jihye Ryu, Hye-Jin Kim, Haeng Eun Song, Jungeun Choi, Gyu-Ho Lee, Sang-Yeob Kim, Song Hwa Park, Dae Gyu Kim, Nam Hoon Kwon, Tai Young Kim, Jean Paul Thiery, Sunghoon Kim, Jung Weon Lee

Present: Due to a technical error during publication, duplicate videos were uploaded as Supplementary File 5 and Supplementary File 28. As a result, Figure 5E contains a reference to the duplicate files.

Corrected: Correct Supplementary File 28 was uploaded. Updated figure 5E can be found below. The publisher apologizes for this oversight.

Original Article: Oncotarget. 2015; 6: 21655-21674. doi: 10.18632/oncotarget.4130.

**Figure 5:**
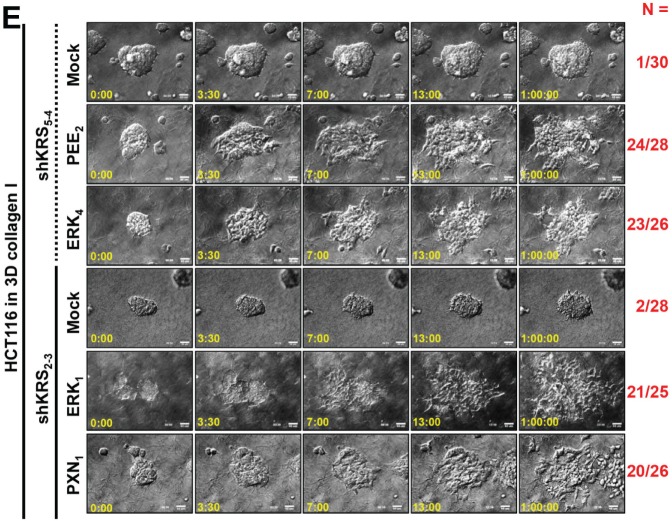
Blockade of dissemination of KRS-suppressed cells was relieved by ERK1/2 and/or paxillin expression (A) HCT116 parental cells were suspended in serum-free BSA containing media, preincubated with normal IgG or functional anti-human integrin α6 blocking antibody (20 μg/ml) for 30 min, and then kept in suspension or reseeded onto laminin-precoated culture dishes with 2% serum-containing media for 2 or 24 h. Whole-cell lysates were immunoblotted for the indicated molecules. (B and C) Cells were transduced with adenovirus (Ad-HA) for R454 (kinase dead) FAK, ΔN(1-100) FAK, or FAK WT for 12 h. The cells were embedded in 3D collagen I gels and, 3 h later, the cell lysates were prepared for immunoblotting (B) or time-lapse imaging was performed for a further 28 h (1:04:00, C). The numbers to the right of the figure in the ‘a/b’ format depict disseminative phenotype cases/total experimental cases. See also Movie S17 to 24. In anti-HA antibody blot, the exogenous FAK showed two different sizes due to WT and its N-terminal deletion, but FAK phosphorylation blots appeared to be single band due to robust phosphorylations mediated by the N-terminal deletion mutant FAK [29]. (D and E) KRS-expressing cells and KRS-suppressed HCT116 cell spheroids (2-1, 2-3 and 5-4 clones) with stable transfection of ERK1/2 (ERK), paxillin (PXN), or ERKs/paxillin (PEE) were embedded in 3D collagen I gels for 24 h, prior to harvesting whole-cell lysates for immunoblottings (D). Alternatively, the spheroids were embedded for 3 h and then time-lapse imaged for another 24 h (E). See also Movie S25 to 30. Data represent three independent experiments.

## SUPPLEMENTARY MATERIAL MOVIE



