# Supplementary material for: Noncanonical roles of membranous lysyl-tRNA synthetase in transducing cell-substrate signaling for invasive dissemination of colon cancer spheroids in 3D collagen I gels
Source: Oncotarget. 2015 May 12;6(25):21655–74. doi: 10.18632/oncotarget.4130 (PMC4673294; doi:10.18632/oncotarget.4130)
Supplement: Supplementary file 1 [file oncotarget-06-21655-s001.pdf]

Noncanonical roles of membranous lysyl-tRNA synthetase in transducing cell-substrate signaling for invasive dissemination of colon cancer spheroids in 3D collagen I gels

Supplementary Material

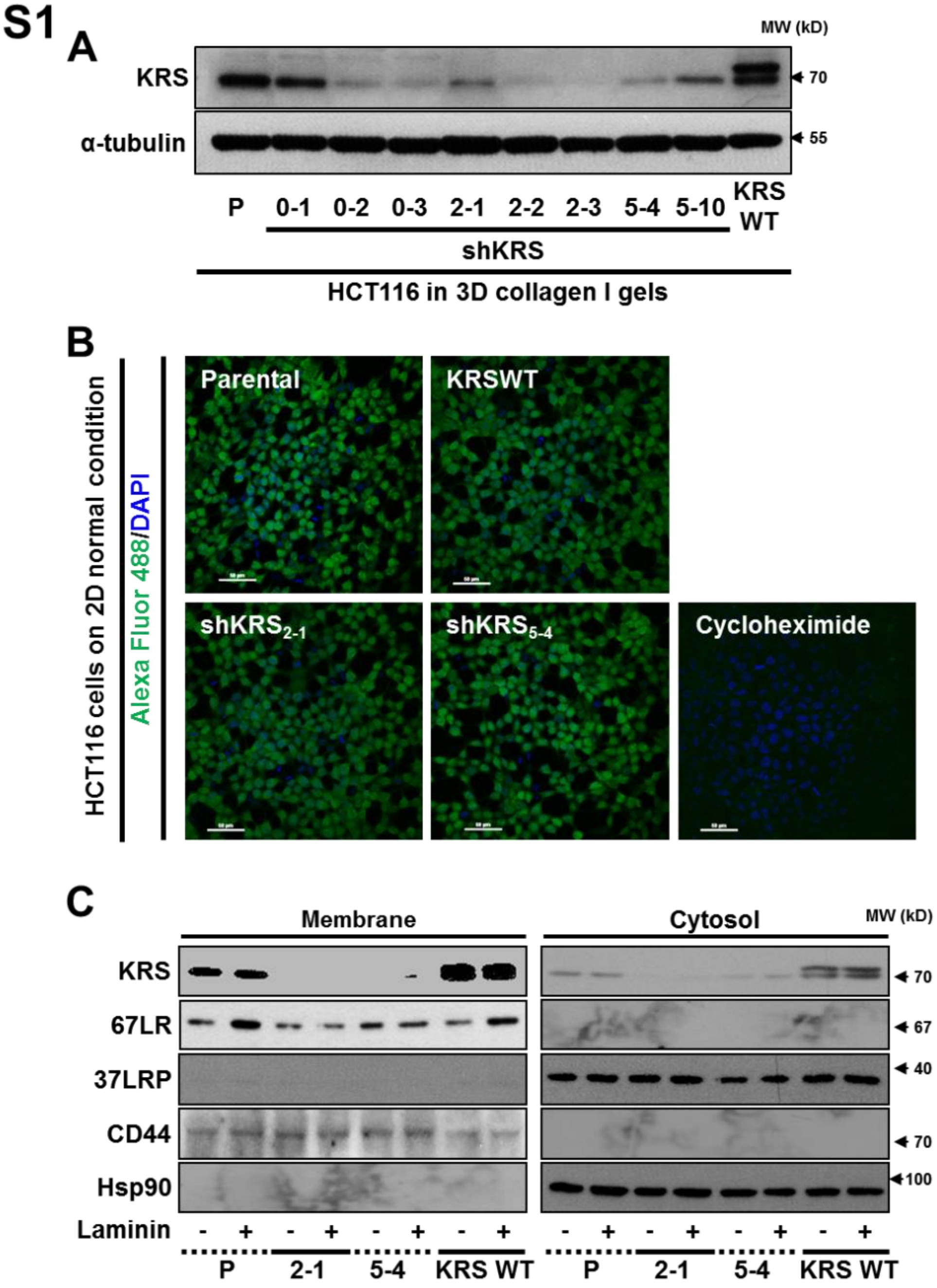

Fig. S1: HCT116 cell clones with various KRS expression levels showed comparable protein synthesis activities. Related to Figure 1. (A) HCT116 parental (P), HCT116 cells stably transfected with shKRS (clones of 0-1, 0-2, 0-3, 2-1, 2-2, 2-3, 5-4, and 5-10), and myc-KRS-overexpressing cells (KRS WT) were harvested for standard western blot analysis, using an antibody against KRS and α-tubulin (loading control). **(B)** General protein synthesis activities among the HCT116 cells, with various KRS expression in normal culture condition without or with cycloheximide (100 μg/ml) for 30 min, were analyzed using the Click-iT® HPG Alexa Fluor® Protein Synthesis Assay Kits (Molecular Probes, Carlsbad, CA). HCT116 cells were analyzed for protein synthesis activity. Cells were cultured with Click-iT® HPG (L-homopropargylglycine, an amino acid analog of methionine) for 30 min without or with cycloheximide (100 μg/ml), and then assessed for the incorporation of the amino acid into proteins, as visualized by green fluorescence for protein synthesis; the assay was performed according to the Click-iT® HPG Alexa Fluor® protein synthesis kit. **(C)** Cells were treated with vehicle PBS or laminin (10 μg/ml) for 1 h in media containing 2% FBS. Cells were then subjected to membrane and cytosol fractionation using Subcellular Proteome Extraction Kit (Calbiochem, Billerica, MA), as indicated by the manusfacturer. HCT116 parental (P), HCT116 cells stably transfected with shKRS (clones of 2-1 or 5-4), and myc-KRS-overexpressing cells (KRS WT) were treated with vehicle PBS or laminin (10 μg/ml) for 1 h in media containing 2% FBS and then harvested for membrane and cytosol fractions. The cell fractions were normalized for standard western blot analysis, and probed using antibodies against the indicated molecules. The data shown represent three independent experiments.

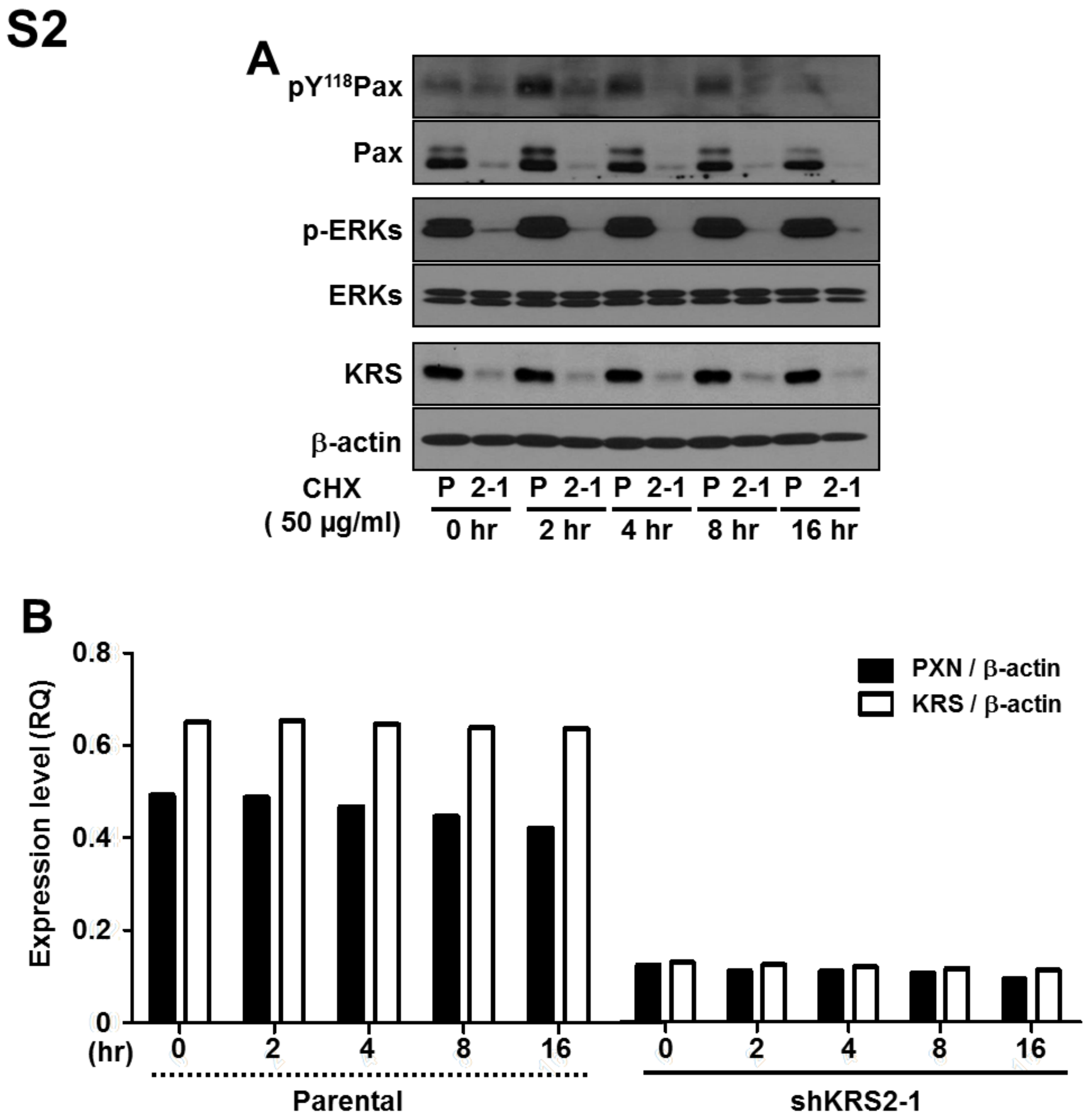

Fig. S2: Paxillin protein stabilities in KRS-expressing parental or KRS-suppressed HCT116 cells. Subconfluent (Parental HCT116 or HCT116-shKRS$_{2-1}$) cells were treated with cycloheximide (CHX, 50 μg/ml) for different periods. After the incubations, cells were harvested for whole cell lysates and the lysates were normalized and preceded for western blots for the indicated molecules (**A**). The band intensities (RQ, relative quantity) for paxillin, KRS, or β-actin were quantitated using GeneTools (SYNGENE, Frederick, MD, USA) image analysis software, and the intensity values for paxillin or KRS were divided by those for β-actin in each condition for graphic presentations **(B).**

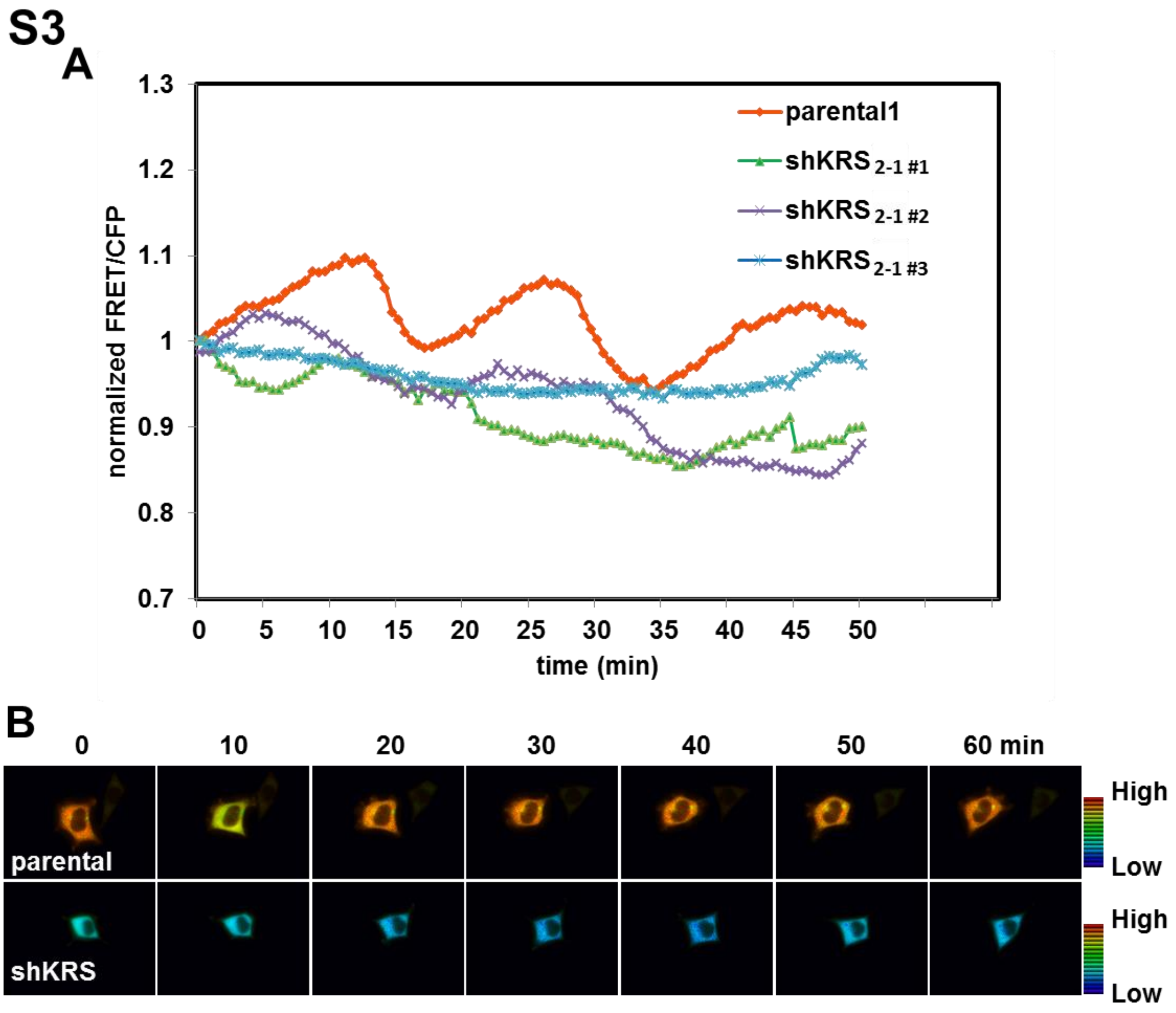

Fig. S3: Oscillation of KRS-dependent ERK activity. Related to Figure 3. Cells replated on laminin (10 μg/ml) were live-imaged for the FRET signal over CFP to support the ERK activity. Whereas KRS-expressing parental HCT116 cells showed an oscillation of ERK activation, KRS-suppressed cells showed gradual signal decline.

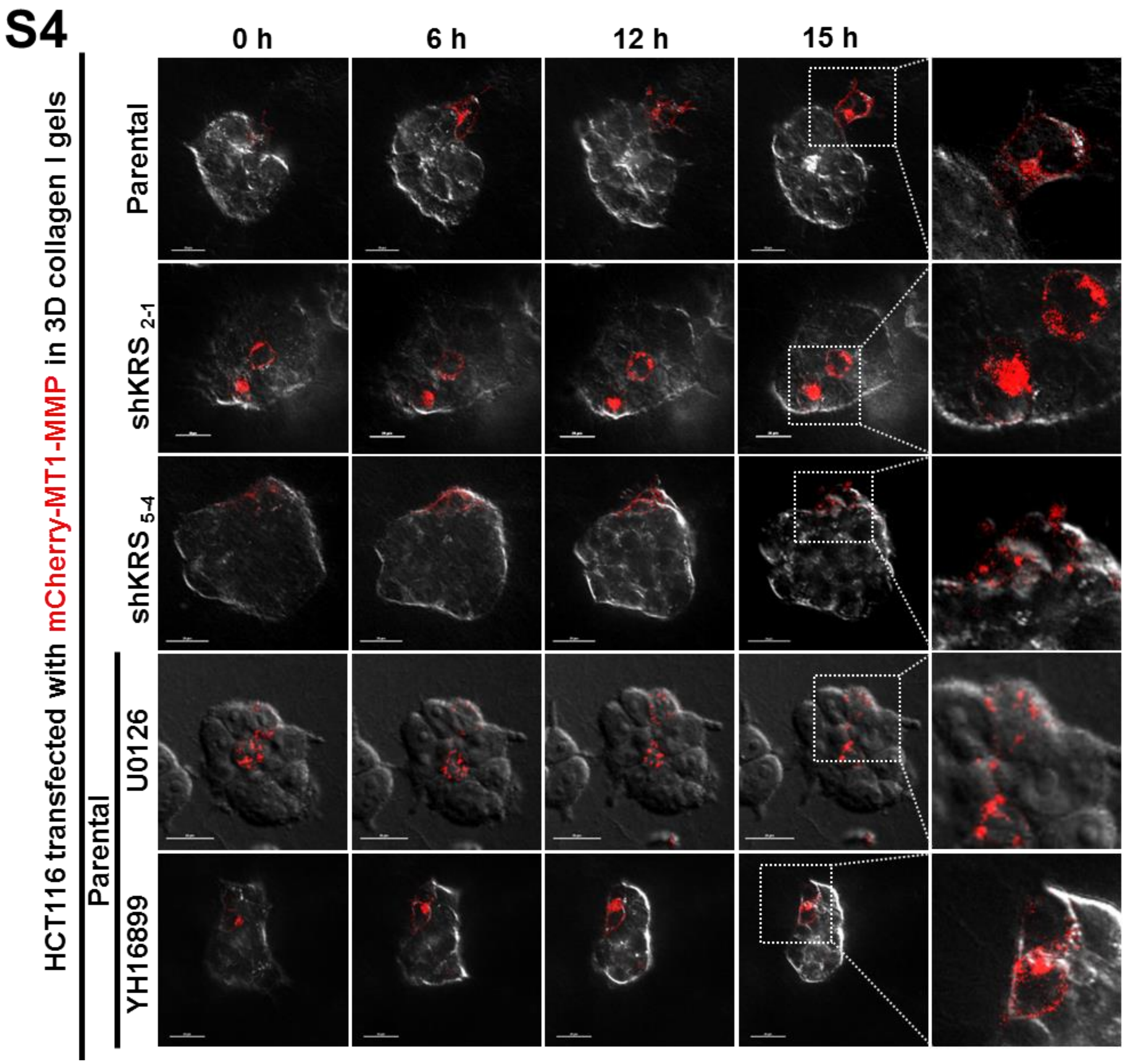

Fig. S4: MT1-MMP appeared not to be significantly involved in the KRS-mediated dissemination from HCT116 colon cancer spheroids in 3D collagen I gels. HCT116 cells transiently transfected with mCherry-conjugated MT1-MMP (red) were embedded into 3D collagen I for 3 h and then live-imaged for dissemination in the absence or presence of U0126 or YH16899 treatment for 15 h.

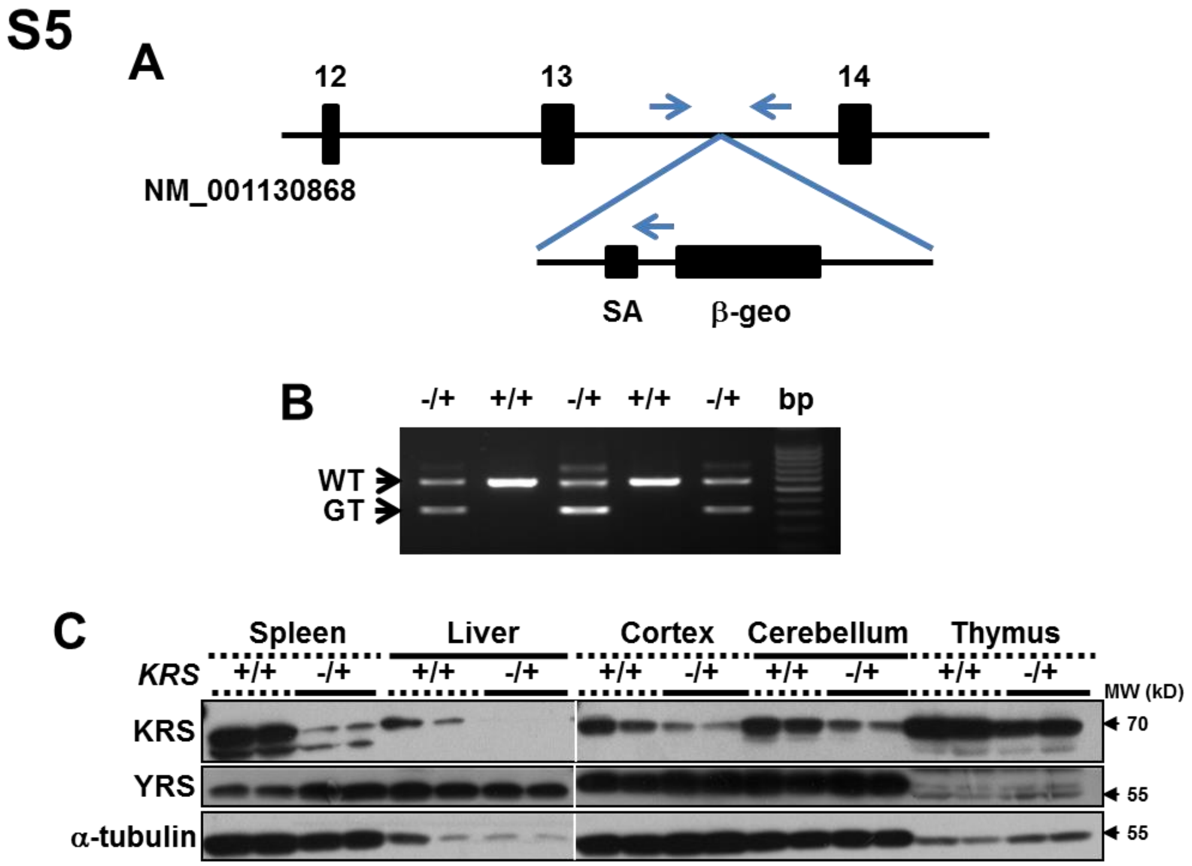

Fig. S5: Generation of KRS heterozygosity in MMTV-PyVT mouse model. Related to Figure 6. **(A)** Genetrap cassette was inserted into intron 13 to disrupt the gene expression of KRS (KARS, NM-001130868). **(B)** KRS expression was compared between two groups [wildtype (WT) versus heterozygous (GT)] by RT-PCR of *KRS* in the primary breast tumor lysate. **(C)** Comparison of KRS immunoblotting in various organs between wild-types and heterozygotes demonstrated the overall reduction of KRS.

Supplementary Movies spread sheets

Supplementary Movie S1: HCT116 parental spheroids embedded in 3D collagen I (2 mg/ml) gels (Related Fig. 1A). Images were analyzed by time-lapse confocal microscopy using a laser-scanning confocal microscope (IX81-ZDC; Olympus). Frames were taken every minute for 30 min.

Supplementary Movie S2: HCT116 $shRNA_{2-1}$ clone spheroids embedded in 3D collagen I (2 mg/ml) gels (Related Fig. 1A). Images were analyzed by time-lapse confocal microscopy using a laser-scanning confocal microscope (IX81-ZDC; Olympus). Frames were taken every minute for 30 min.

Supplementary Movie S3: HCT116 $shRNA_{2-2}$ clone spheroids embedded in 3D collagen I (2 mg/ml) gels (Related Fig. 1A). Images were analyzed by time-lapse confocal microscopy using a laser-scanning confocal microscope (IX81-ZDC; Olympus). Frames were taken every minute for 30 min.

Supplementary Movie S4: HCT116 $shRNA_{2-3}$ clone spheroids embedded in 3D collagen I (2 mg/ml) gels (Related Fig. 1A). Images were analyzed by time-lapse confocal microscopy using a laser-scanning confocal microscope (IX81-ZDC; Olympus). Frames were taken every minute for 30 min.

Supplementary Movie S5: HCT116 $shRNA_{5-4}$ clone spheroids embedded in 3D collagen I (2 mg/ml) gels (Related Fig. 1A). Images were analyzed by time-lapse confocal microscopy using a laser-scanning confocal microscope (IX81-ZDC; Olympus). Frames were taken every minute for 30 min.

Supplementary Movie S6: HCT116 myc-KRS WT clone spheroids embedded in 3D collagen I (2 mg/ml) gels (Related Fig. 1A). Images were analyzed by time-lapse confocal microscopy using a laser-scanning confocal microscope (IX81-ZDC; Olympus). Frames were taken every minute for 30 min.

Supplementary Movie S7: SW620 parental spheroids stably transfected with shControl embedded in 3D collagen I (2 mg/ml) gels (Related Fig. 1B). Images were analyzed by time-lapse confocal microscopy using a laser-scanning confocal microscope (IX81-ZDC; Olympus). Frames were taken every minute for 30 min.

Supplementary Movie S8: SW620 parental spheroids stably transfected with $shKRS_{\#2}$ embedded in 3D collagen I (2 mg/ml) gels (Related Fig. 1B). Images were analyzed by time-lapse confocal microscopy using a laser-scanning confocal microscope (IX81-ZDC; Olympus). Frames were taken every minute for 30 min.

Supplementary Movie S9: SW620 parental spheroids stably transfected with $shKRS_{\#5}$ embedded in 3D collagen I (2 mg/ml) gels (Related Fig. 1B). Images were analyzed by time-lapse confocal microscopy using a laser-scanning confocal microscope (IX81-ZDC; Olympus). Frames were taken every minute for 30 min.

Supplementary Movie S10: FRET signaling for ERK activity of HCT116 parental on 2D laminin using a Nikon Ti-E inverted microscope (Nikon, Tokyo, Japan) (Related Fig. S2B). Frames were taken every minute for 30 sec.

Supplementary Movie S11: FRET signaling for ERK activity of HCT116 $shKRS_{2-1}$ on 2D laminin using a Nikon Ti-E inverted microscope (Nikon, Tokyo, Japan) (Related Fig. S2B). Frames were taken every minute for 30 sec.

Supplementary Movie S12: HCT116 parental control spheroids embedded in 3D collagen I (2 mg/ml) gels (Related Fig. 4A). Images were analyzed by time-lapse confocal microscopy

using a laser-scanning confocal microscope (IX81-ZDC; Olympus). Frames were taken every minute for 30 min.

Supplementary Movie S13: HCT116 parental spheroids embedded in 3D collagen I (2 mg/ml) gels in the presence of 50 μM U0126 treatment (Related Fig. 4A). Images were analyzed by time-lapse confocal microscopy using a laser-scanning confocal microscope (IX81-ZDC; Olympus). Frames were taken every minute for 30 min.

Supplementary Movie S14: HCT116 parental spheroids embedded in 3D collagen I (2 mg/ml) gels in the presence of 50 μM YH16899 treatment (Related Fig. 4A). Images were analyzed by time-lapse confocal microscopy using a laser-scanning confocal microscope (IX81-ZDC; Olympus). Frames were taken every minute for 30 min.

Supplementary Movie S15: SW620 parental spheroids embedded in 3D collagen I (2 mg/ml) gels in the presence of 50 μM U0126 treatment (Related Fig. 4A). Images were analyzed by time-lapse confocal microscopy using a laser-scanning confocal microscope (IX81-ZDC; Olympus). Frames were taken every minute for 30 min

Supplementary Movie S16: SW620 parental spheroids embedded in 3D collagen I (2 mg/ml) gels in the presence of 50 μM YH16899 treatment (Related Fig. 4A). Images were analyzed by time-lapse confocal microscopy using a laser-scanning confocal microscope (IX81-ZDC; Olympus). Frames were taken every minute for 30 min.

Supplementary Movie S17: HCT116 parental spheroids transduced with control Ad-HA adenovirus embedded in 3D collagen I (2 mg/ml) gels (Related Fig. 5C). Images were analyzed by time-lapse confocal microscopy using a laser-scanning confocal microscope (IX81-ZDC; Olympus). Frames were taken every minute for 30 min.

Supplementary Movie S18. HCT116 parental spheroids transduced with control Ad-HA-R454 FA adenovirus embedded in 3D collagen I (2 mg/ml) gels (Related Fig. 5C). Images were analyzed by time-lapse confocal microscopy using a laser-scanning confocal microscope (IX81-ZDC; Olympus). Frames were taken every minute for 30 min.

Supplementary Movie S19: HCT116 $shKRS_{2\text{-}1}$ spheroids transduced with control Ad-HA adenovirus embedded in 3D collagen I (2 mg/ml) gels (Related Fig. 5C). Images were analyzed by time-lapse confocal microscopy using a laser-scanning confocal microscope (IX81-ZDC; Olympus). Frames were taken every minute for 30 min.

Supplementary Movie S20: HCT116 $shKRS_{2\text{-}1}$ spheroids transduced with control Ad-HA-ΔNFAK adenovirus embedded in 3D collagen I (2 mg/ml) gels (Related Fig. 5C). Images were analyzed by time-lapse confocal microscopy using a laser-scanning confocal microscope (IX81-ZDC; Olympus). Frames were taken every minute for 30 min.

Supplementary Movie S21: HCT116 $shKRS_{2\text{-}1}$ spheroids transduced with control Ad-HA FAK-WT adenovirus embedded in 3D collagen I (2 mg/ml) gels (Related Fig. 5C). Images were analyzed by time-lapse confocal microscopy using a laser-scanning confocal microscope (IX81-ZDC; Olympus). Frames were taken every minute for 30 min.

Supplementary Movie S22: HCT116 $shKRS_{5\text{-}4}$ spheroids transduced with control Ad-HA adenovirus embedded in 3D collagen I (2 mg/ml) gels (Related Fig. 5C). Images were analyzed by time-lapse confocal microscopy using a laser-scanning confocal microscope (IX81-ZDC; Olympus). Frames were taken every minute for 30 min.

Supplementary Movie S23: HCT116 $shKRS_{5\text{-}4}$ spheroids transduced with control Ad-HA-ΔNFAK adenovirus embedded in 3D collagen I (2 mg/ml) gels (Related Fig. 5C). Images were analyzed by time-lapse confocal microscopy using a laser-scanning confocal

microscope (IX81-ZDC; Olympus). Frames were taken every minute for 30 min.

Supplementary Movie S24: HCT116 shKRS$_{5\text{-}4}$ spheroids transduced with control Ad-HA FAK-WT adenovirus embedded in 3D collagen I (2 mg/ml) gels (Related Fig. 5C). Images were analyzed by time-lapse confocal microscopy using a laser-scanning confocal microscope (IX81-ZDC; Olympus). Frames were taken every minute for 30 min.

Supplementary Movie S25: HCT116 shKRS$_{5\text{-}4}$ spheroids stably transfected with mock plasmid and embedded in 3D collagen I (2 mg/ml) gels (Related Fig. 5E). Images were analyzed by time-lapse confocal microscopy using a laser-scanning confocal microscope (IX81-ZDC; Olympus). Frames were taken every minute for 30 min.

Supplementary Movie S26: HCT116 shKRS$_{5\text{-}4}$ spheroids stably transfected with paxillin, ERK1, and ERK2 and embedded in 3D collagen I (2 mg/ml) gels (Related Fig. 5E). Images were analyzed by time-lapse confocal microscopy using a laser-scanning confocal microscope (IX81-ZDC; Olympus). Frames were taken every minute for 30 min.

Supplementary Movie S27: HCT116 shKRS$_{5\text{-}4}$ spheroids stably transfected with ERK1 and embedded in 3D collagen I (2 mg/ml) gels (Related Fig. 5E). Images were analyzed by time-lapse confocal microscopy using a laser-scanning confocal microscope (IX81-ZDC; Olympus). Frames were taken every minute for 30 min.

Supplementary Movie S28. HCT116 shKRS$_{2\text{-}3}$ spheroids stably transfected with mock plasmid and embedded in 3D collagen I (2 mg/ml) gels (Related Fig. 5E). Images were analyzed by time-lapse confocal microscopy using a laser-scanning confocal microscope (IX81-ZDC; Olympus). Frames were taken every minute for 30 min.

Supplementary Movie S29: HCT116 shKRS$_{2\text{-}3}$ spheroids stably transfected with ERK1 and embedded in 3D collagen I (2 mg/ml) gels (Related Fig. 5E). Images were analyzed by time-lapse confocal microscopy using a laser-scanning confocal microscope (IX81-ZDC; Olympus). Frames were taken every minute for 30 min.

Supplementary Movie S30: HCT116 shKRS$_{2\text{-}3}$ spheroids stably transfected with paxillin and embedded in 3D collagen I (2 mg/ml) gels (Related Fig. 5E). Images were analyzed by time-lapse confocal microscopy using a laser-scanning confocal microscope (IX81-ZDC; Olympus). Frames were taken every minute for 30 min.
